# Study of the spin and parity of the Higgs boson in diboson decays with the ATLAS detector

**DOI:** 10.1140/epjc/s10052-015-3685-1

**Published:** 2015-10-06

**Authors:** G. Aad, B. Abbott, J. Abdallah, O. Abdinov, R. Aben, M. Abolins, O. S. AbouZeid, H. Abramowicz, H. Abreu, R. Abreu, Y. Abulaiti, B. S. Acharya, L. Adamczyk, D. L. Adams, J. Adelman, S. Adomeit, T. Adye, A. A. Affolder, T. Agatonovic-Jovin, J. Agricola, J. A. Aguilar-Saavedra, S. P. Ahlen, F. Ahmadov, G. Aielli, H. Akerstedt, T. P. A. Åkesson, A. V. Akimov, G. L. Alberghi, J. Albert, S. Albrand, M. J. Alconada Verzini, M. Aleksa, I. N. Aleksandrov, C. Alexa, G. Alexander, T. Alexopoulos, M. Alhroob, G. Alimonti, L. Alio, J. Alison, S. P. Alkire, B. M. M. Allbrooke, P. P. Allport, A. Aloisio, A. Alonso, F. Alonso, C. Alpigiani, A. Altheimer, B. Alvarez Gonzalez, D. Álvarez Piqueras, M. G. Alviggi, B. T. Amadio, K. Amako, Y. Amaral Coutinho, C. Amelung, D. Amidei, S. P. Amor Dos Santos, A. Amorim, S. Amoroso, N. Amram, G. Amundsen, C. Anastopoulos, L. S. Ancu, N. Andari, T. Andeen, C. F. Anders, G. Anders, J. K. Anders, K. J. Anderson, A. Andreazza, V. Andrei, S. Angelidakis, I. Angelozzi, P. Anger, A. Angerami, F. Anghinolfi, A. V. Anisenkov, N. Anjos, A. Annovi, M. Antonelli, A. Antonov, J. Antos, F. Anulli, M. Aoki, L. Aperio Bella, G. Arabidze, Y. Arai, J. P. Araque, A. T. H. Arce, F. A. Arduh, J-F. Arguin, S. Argyropoulos, M. Arik, A. J. Armbruster, O. Arnaez, V. Arnal, H. Arnold, M. Arratia, O. Arslan, A. Artamonov, G. Artoni, S. Asai, N. Asbah, A. Ashkenazi, B. Åsman, L. Asquith, K. Assamagan, R. Astalos, M. Atkinson, N. B. Atlay, B. Auerbach, K. Augsten, M. Aurousseau, G. Avolio, B. Axen, M. K. Ayoub, G. Azuelos, M. A. Baak, A. E. Baas, C. Bacci, H. Bachacou, K. Bachas, M. Backes, M. Backhaus, P. Bagiacchi, P. Bagnaia, Y. Bai, T. Bain, J. T. Baines, O. K. Baker, E. M. Baldin, P. Balek, T. Balestri, F. Balli, E. Banas, Sw. Banerjee, A. A. E. Bannoura, H. S. Bansil, L. Barak, E. L. Barberio, D. Barberis, M. Barbero, T. Barillari, M. Barisonzi, T. Barklow, N. Barlow, S. L. Barnes, B. M. Barnett, R. M. Barnett, Z. Barnovska, A. Baroncelli, G. Barone, A. J. Barr, F. Barreiro, J. Barreiro Guimarães da Costa, R. Bartoldus, A. E. Barton, P. Bartos, A. Basalaev, A. Bassalat, A. Basye, R. L. Bates, S. J. Batista, J. R. Batley, M. Battaglia, M. Bauce, F. Bauer, H. S. Bawa, J. B. Beacham, M. D. Beattie, T. Beau, P. H. Beauchemin, R. Beccherle, P. Bechtle, H. P. Beck, K. Becker, M. Becker, S. Becker, M. Beckingham, C. Becot, A. J. Beddall, A. Beddall, V. A. Bednyakov, C. P. Bee, L. J. Beemster, T. A. Beermann, M. Begel, J. K. Behr, C. Belanger-Champagne, W. H. Bell, G. Bella, L. Bellagamba, A. Bellerive, M. Bellomo, K. Belotskiy, O. Beltramello, O. Benary, D. Benchekroun, M. Bender, K. Bendtz, N. Benekos, Y. Benhammou, E. Benhar Noccioli, J. A. Benitez Garcia, D. P. Benjamin, J. R. Bensinger, S. Bentvelsen, L. Beresford, M. Beretta, D. Berge, E. Bergeaas Kuutmann, N. Berger, F. Berghaus, J. Beringer, C. Bernard, N. R. Bernard, C. Bernius, F. U. Bernlochner, T. Berry, P. Berta, C. Bertella, G. Bertoli, F. Bertolucci, C. Bertsche, D. Bertsche, M. I. Besana, G. J. Besjes, O. Bessidskaia Bylund, M. Bessner, N. Besson, C. Betancourt, S. Bethke, A. J. Bevan, W. Bhimji, R. M. Bianchi, L. Bianchini, M. Bianco, O. Biebel, D. Biedermann, S. P. Bieniek, M. Biglietti, J. Bilbao De Mendizabal, H. Bilokon, M. Bindi, S. Binet, A. Bingul, C. Bini, S. Biondi, C. W. Black, J. E. Black, K. M. Black, D. Blackburn, R. E. Blair, J.-B. Blanchard, J. E. Blanco, T. Blazek, I. Bloch, C. Blocker, W. Blum, U. Blumenschein, G. J. Bobbink, V. S. Bobrovnikov, S. S. Bocchetta, A. Bocci, C. Bock, M. Boehler, J. A. Bogaerts, D. Bogavac, A. G. Bogdanchikov, C. Bohm, V. Boisvert, T. Bold, V. Boldea, A. S. Boldyrev, M. Bomben, M. Bona, M. Boonekamp, A. Borisov, G. Borissov, S. Borroni, J. Bortfeldt, V. Bortolotto, K. Bos, D. Boscherini, M. Bosman, J. Boudreau, J. Bouffard, E. V. Bouhova-Thacker, D. Boumediene, C. Bourdarios, N. Bousson, A. Boveia, J. Boyd, I. R. Boyko, I. Bozic, J. Bracinik, A. Brandt, G. Brandt, O. Brandt, U. Bratzler, B. Brau, J. E. Brau, H. M. Braun, S. F. Brazzale, W. D. Breaden Madden, K. Brendlinger, A. J. Brennan, L. Brenner, R. Brenner, S. Bressler, K. Bristow, T. M. Bristow, D. Britton, D. Britzger, F. M. Brochu, I. Brock, R. Brock, J. Bronner, G. Brooijmans, T. Brooks, W. K. Brooks, J. Brosamer, E. Brost, J. Brown, P. A. Bruckman de Renstrom, D. Bruncko, R. Bruneliere, A. Bruni, G. Bruni, M. Bruschi, N. Bruscino, L. Bryngemark, T. Buanes, Q. Buat, P. Buchholz, A. G. Buckley, S. I. Buda, I. A. Budagov, F. Buehrer, L. Bugge, M. K. Bugge, O. Bulekov, D. Bullock, H. Burckhart, S. Burdin, B. Burghgrave, S. Burke, I. Burmeister, E. Busato, D. Büscher, V. Büscher, P. Bussey, J. M. Butler, A. I. Butt, C. M. Buttar, J. M. Butterworth, P. Butti, W. Buttinger, A. Buzatu, A. R. Buzykaev, S. Cabrera Urbán, D. Caforio, V. M. Cairo, O. Cakir, P. Calafiura, A. Calandri, G. Calderini, P. Calfayan, L. P. Caloba, D. Calvet, S. Calvet, R. Camacho Toro, S. Camarda, P. Camarri, D. Cameron, R. Caminal Armadans, S. Campana, M. Campanelli, A. Campoverde, V. Canale, A. Canepa, M. Cano Bret, J. Cantero, R. Cantrill, T. Cao, M. D. M. Capeans Garrido, I. Caprini, M. Caprini, M. Capua, R. Caputo, R. Cardarelli, F. Cardillo, T. Carli, G. Carlino, L. Carminati, S. Caron, E. Carquin, G. D. Carrillo-Montoya, J. R. Carter, J. Carvalho, D. Casadei, M. P. Casado, M. Casolino, E. Castaneda-Miranda, A. Castelli, V. Castillo Gimenez, N. F. Castro, P. Catastini, A. Catinaccio, J. R. Catmore, A. Cattai, J. Caudron, V. Cavaliere, D. Cavalli, M. Cavalli-Sforza, V. Cavasinni, F. Ceradini, B. C. Cerio, K. Cerny, A. S. Cerqueira, A. Cerri, L. Cerrito, F. Cerutti, M. Cerv, A. Cervelli, S. A. Cetin, A. Chafaq, D. Chakraborty, I. Chalupkova, P. Chang, J. D. Chapman, D. G. Charlton, C. C. Chau, C. A. Chavez Barajas, S. Cheatham, A. Chegwidden, S. Chekanov, S. V. Chekulaev, G. A. Chelkov, M. A. Chelstowska, C. Chen, H. Chen, K. Chen, L. Chen, S. Chen, X. Chen, Y. Chen, H. C. Cheng, Y. Cheng, A. Cheplakov, E. Cheremushkina, R. Cherkaoui El Moursli, V. Chernyatin, E. Cheu, L. Chevalier, V. Chiarella, J. T. Childers, G. Chiodini, A. S. Chisholm, R. T. Chislett, A. Chitan, M. V. Chizhov, K. Choi, S. Chouridou, B. K. B. Chow, V. Christodoulou, D. Chromek-Burckhart, J. Chudoba, A. J. Chuinard, J. J. Chwastowski, L. Chytka, G. Ciapetti, A. K. Ciftci, D. Cinca, V. Cindro, I. A. Cioara, A. Ciocio, F. Cirotto, Z. H. Citron, M. Ciubancan, A. Clark, B. L. Clark, P. J. Clark, R. N. Clarke, W. Cleland, C. Clement, Y. Coadou, M. Cobal, A. Coccaro, J. Cochran, L. Coffey, J. G. Cogan, B. Cole, S. Cole, A. P. Colijn, J. Collot, T. Colombo, G. Compostella, P. Conde Muiño, E. Coniavitis, S. H. Connell, I. A. Connelly, S. M. Consonni, V. Consorti, S. Constantinescu, C. Conta, G. Conti, F. Conventi, M. Cooke, B. D. Cooper, A. M. Cooper-Sarkar, T. Cornelissen, M. Corradi, F. Corriveau, A. Corso-Radu, A. Cortes-Gonzalez, G. Cortiana, G. Costa, M. J. Costa, D. Costanzo, D. Côté, G. Cottin, G. Cowan, B. E. Cox, K. Cranmer, G. Cree, S. Crépé-Renaudin, F. Crescioli, W. A. Cribbs, M. Crispin Ortuzar, M. Cristinziani, V. Croft, G. Crosetti, T. Cuhadar Donszelmann, J. Cummings, M. Curatolo, C. Cuthbert, H. Czirr, P. Czodrowski, S. D’Auria, M. D’Onofrio, M. J. Da Cunha Sargedas De Sousa, C. Da Via, W. Dabrowski, A. Dafinca, T. Dai, O. Dale, F. Dallaire, C. Dallapiccola, M. Dam, J. R. Dandoy, N. P. Dang, A. C. Daniells, M. Danninger, M. Dano Hoffmann, V. Dao, G. Darbo, S. Darmora, J. Dassoulas, A. Dattagupta, W. Davey, C. David, T. Davidek, E. Davies, M. Davies, P. Davison, Y. Davygora, E. Dawe, I. Dawson, R. K. Daya-Ishmukhametova, K. De, R. de Asmundis, S. De Castro, S. De Cecco, N. De Groot, P. de Jong, H. De la Torre, F. De Lorenzi, L. De Nooij, D. De Pedis, A. De Salvo, U. De Sanctis, A. De Santo, J. B. De Vivie De Regie, W. J. Dearnaley, R. Debbe, C. Debenedetti, D. V. Dedovich, I. Deigaard, J. Del Peso, T. Del Prete, D. Delgove, F. Deliot, C. M. Delitzsch, M. Deliyergiyev, A. Dell’Acqua, L. Dell’Asta, M. Dell’Orso, M. Della Pietra, D. della Volpe, M. Delmastro, P. A. Delsart, C. Deluca, D. A. DeMarco, S. Demers, M. Demichev, A. Demilly, S. P. Denisov, D. Derendarz, J. E. Derkaoui, F. Derue, P. Dervan, K. Desch, C. Deterre, P. O. Deviveiros, A. Dewhurst, S. Dhaliwal, A. Di Ciaccio, L. Di Ciaccio, A. Di Domenico, C. Di Donato, A. Di Girolamo, B. Di Girolamo, A. Di Mattia, B. Di Micco, R. Di Nardo, A. Di Simone, R. Di Sipio, D. Di Valentino, C. Diaconu, M. Diamond, F. A. Dias, M. A. Diaz, E. B. Diehl, J. Dietrich, S. Diglio, A. Dimitrievska, J. Dingfelder, P. Dita, S. Dita, F. Dittus, F. Djama, T. Djobava, J. I. Djuvsland, M. A. B. do Vale, D. Dobos, M. Dobre, C. Doglioni, T. Dohmae, J. Dolejsi, Z. Dolezal, B. A. Dolgoshein, M. Donadelli, S. Donati, P. Dondero, J. Donini, J. Dopke, A. Doria, M. T. Dova, A. T. Doyle, E. Drechsler, M. Dris, E. Dubreuil, E. Duchovni, G. Duckeck, O. A. Ducu, D. Duda, A. Dudarev, L. Duflot, L. Duguid, M. Dührssen, M. Dunford, H. Duran Yildiz, M. Düren, A. Durglishvili, D. Duschinger, M. Dyndal, C. Eckardt, K. M. Ecker, R. C. Edgar, W. Edson, N. C. Edwards, W. Ehrenfeld, T. Eifert, G. Eigen, K. Einsweiler, T. Ekelof, M. El Kacimi, M. Ellert, S. Elles, F. Ellinghaus, A. A. Elliot, N. Ellis, J. Elmsheuser, M. Elsing, D. Emeliyanov, Y. Enari, O. C. Endner, M. Endo, J. Erdmann, A. Ereditato, G. Ernis, J. Ernst, M. Ernst, S. Errede, E. Ertel, M. Escalier, H. Esch, C. Escobar, B. Esposito, A. I. Etienvre, E. Etzion, H. Evans, A. Ezhilov, L. Fabbri, G. Facini, R. M. Fakhrutdinov, S. Falciano, R. J. Falla, J. Faltova, Y. Fang, M. Fanti, A. Farbin, A. Farilla, T. Farooque, S. Farrell, S. M. Farrington, P. Farthouat, F. Fassi, P. Fassnacht, D. Fassouliotis, M. Faucci Giannelli, A. Favareto, L. Fayard, P. Federic, O. L. Fedin, W. Fedorko, S. Feigl, L. Feligioni, C. Feng, E. J. Feng, H. Feng, A. B. Fenyuk, L. Feremenga, P. Fernandez Martinez, S. Fernandez Perez, J. Ferrando, A. Ferrari, P. Ferrari, R. Ferrari, D. E. Ferreira de Lima, A. Ferrer, D. Ferrere, C. Ferretti, A. Ferretto Parodi, M. Fiascaris, F. Fiedler, A. Filipčič, M. Filipuzzi, F. Filthaut, M. Fincke-Keeler, K. D. Finelli, M. C. N. Fiolhais, L. Fiorini, A. Firan, A. Fischer, C. Fischer, J. Fischer, W. C. Fisher, E. A. Fitzgerald, N. Flaschel, I. Fleck, P. Fleischmann, S. Fleischmann, G. T. Fletcher, G. Fletcher, R. R. M. Fletcher, T. Flick, A. Floderus, L. R. Flores Castillo, M. J. Flowerdew, A. Formica, A. Forti, D. Fournier, H. Fox, S. Fracchia, P. Francavilla, M. Franchini, D. Francis, L. Franconi, M. Franklin, M. Frate, M. Fraternali, D. Freeborn, S. T. French, F. Friedrich, D. Froidevaux, J. A. Frost, C. Fukunaga, E. Fullana Torregrosa, B. G. Fulsom, J. Fuster, C. Gabaldon, O. Gabizon, A. Gabrielli, A. Gabrielli, S. Gadatsch, S. Gadomski, G. Gagliardi, P. Gagnon, C. Galea, B. Galhardo, E. J. Gallas, B. J. Gallop, P. Gallus, G. Galster, K. K. Gan, J. Gao, Y. Gao, Y. S. Gao, F. M. Garay Walls, F. Garberson, C. García, J. E. García Navarro, M. Garcia-Sciveres, R. W. Gardner, N. Garelli, V. Garonne, C. Gatti, A. Gaudiello, G. Gaudio, B. Gaur, L. Gauthier, P. Gauzzi, I. L. Gavrilenko, C. Gay, G. Gaycken, E. N. Gazis, P. Ge, Z. Gecse, C. N. P. Gee, D. A. A. Geerts, Ch. Geich-Gimbel, M. P. Geisler, C. Gemme, M. H. Genest, S. Gentile, M. George, S. George, D. Gerbaudo, A. Gershon, S. Ghasemi, H. Ghazlane, B. Giacobbe, S. Giagu, V. Giangiobbe, P. Giannetti, B. Gibbard, S. M. Gibson, M. Gilchriese, T. P. S. Gillam, D. Gillberg, G. Gilles, D. M. Gingrich, N. Giokaris, M. P. Giordani, F. M. Giorgi, F. M. Giorgi, P. F. Giraud, P. Giromini, D. Giugni, C. Giuliani, M. Giulini, B. K. Gjelsten, S. Gkaitatzis, I. Gkialas, E. L. Gkougkousis, L. K. Gladilin, C. Glasman, J. Glatzer, P. C. F. Glaysher, A. Glazov, M. Goblirsch-Kolb, J. R. Goddard, J. Godlewski, S. Goldfarb, T. Golling, D. Golubkov, A. Gomes, R. Gonçalo, J. Goncalves Pinto Firmino Da Costa, L. Gonella, S. González de la Hoz, G. Gonzalez Parra, S. Gonzalez-Sevilla, L. Goossens, P. A. Gorbounov, H. A. Gordon, I. Gorelov, B. Gorini, E. Gorini, A. Gorišek, E. Gornicki, A. T. Goshaw, C. Gössling, M. I. Gostkin, D. Goujdami, A. G. Goussiou, N. Govender, E. Gozani, H. M. X. Grabas, L. Graber, I. Grabowska-Bold, P. Grafström, K-J. Grahn, J. Gramling, E. Gramstad, S. Grancagnolo, V. Grassi, V. Gratchev, H. M. Gray, E. Graziani, Z. D. Greenwood, K. Gregersen, I. M. Gregor, P. Grenier, J. Griffiths, A. A. Grillo, K. Grimm, S. Grinstein, Ph. Gris, J.-F. Grivaz, J. P. Grohs, A. Grohsjean, E. Gross, J. Grosse-Knetter, G. C. Grossi, Z. J. Grout, L. Guan, J. Guenther, F. Guescini, D. Guest, O. Gueta, E. Guido, T. Guillemin, S. Guindon, U. Gul, C. Gumpert, J. Guo, Y. Guo, S. Gupta, G. Gustavino, P. Gutierrez, N. G. Gutierrez Ortiz, C. Gutschow, C. Guyot, C. Gwenlan, C. B. Gwilliam, A. Haas, C. Haber, H. K. Hadavand, N. Haddad, P. Haefner, S. Hageböck, Z. Hajduk, H. Hakobyan, M. Haleem, J. Haley, D. Hall, G. Halladjian, G. D. Hallewell, K. Hamacher, P. Hamal, K. Hamano, M. Hamer, A. Hamilton, G. N. Hamity, P. G. Hamnett, L. Han, K. Hanagaki, K. Hanawa, M. Hance, P. Hanke, R. Hanna, J. B. Hansen, J. D. Hansen, M. C. Hansen, P. H. Hansen, K. Hara, A. S. Hard, T. Harenberg, F. Hariri, S. Harkusha, R. D. Harrington, P. F. Harrison, F. Hartjes, M. Hasegawa, S. Hasegawa, Y. Hasegawa, A. Hasib, S. Hassani, S. Haug, R. Hauser, L. Hauswald, M. Havranek, C. M. Hawkes, R. J. Hawkings, A. D. Hawkins, T. Hayashi, D. Hayden, C. P. Hays, J. M. Hays, H. S. Hayward, S. J. Haywood, S. J. Head, T. Heck, V. Hedberg, L. Heelan, S. Heim, T. Heim, B. Heinemann, L. Heinrich, J. Hejbal, L. Helary, S. Hellman, D. Hellmich, C. Helsens, J. Henderson, R. C. W. Henderson, Y. Heng, C. Hengler, A. Henrichs, A. M. Henriques Correia, S. Henrot-Versille, G. H. Herbert, Y. Hernández Jiménez, R. Herrberg-Schubert, G. Herten, R. Hertenberger, L. Hervas, G. G. Hesketh, N. P. Hessey, J. W. Hetherly, R. Hickling, E. Higón-Rodriguez, E. Hill, J. C. Hill, K. H. Hiller, S. J. Hillier, I. Hinchliffe, E. Hines, R. R. Hinman, M. Hirose, D. Hirschbuehl, J. Hobbs, N. Hod, M. C. Hodgkinson, P. Hodgson, A. Hoecker, M. R. Hoeferkamp, F. Hoenig, M. Hohlfeld, D. Hohn, T. R. Holmes, M. Homann, T. M. Hong, L. Hooft van Huysduynen, W. H. Hopkins, Y. Horii, A. J. Horton, J-Y. Hostachy, S. Hou, A. Hoummada, J. Howard, J. Howarth, M. Hrabovsky, I. Hristova, J. Hrivnac, T. Hryn’ova, A. Hrynevich, C. Hsu, P. J. Hsu, S.-C. Hsu, D. Hu, Q. Hu, X. Hu, Y. Huang, Z. Hubacek, F. Hubaut, F. Huegging, T. B. Huffman, E. W. Hughes, G. Hughes, M. Huhtinen, T. A. Hülsing, N. Huseynov, J. Huston, J. Huth, G. Iacobucci, G. Iakovidis, I. Ibragimov, L. Iconomidou-Fayard, E. Ideal, Z. Idrissi, P. Iengo, O. Igonkina, T. Iizawa, Y. Ikegami, K. Ikematsu, M. Ikeno, Y. Ilchenko, D. Iliadis, N. Ilic, T. Ince, G. Introzzi, P. Ioannou, M. Iodice, K. Iordanidou, V. Ippolito, A. Irles Quiles, C. Isaksson, M. Ishino, M. Ishitsuka, R. Ishmukhametov, C. Issever, S. Istin, J. M. Iturbe Ponce, R. Iuppa, J. Ivarsson, W. Iwanski, H. Iwasaki, J. M. Izen, V. Izzo, S. Jabbar, B. Jackson, M. Jackson, P. Jackson, M. R. Jaekel, V. Jain, K. Jakobs, S. Jakobsen, T. Jakoubek, J. Jakubek, D. O. Jamin, D. K. Jana, E. Jansen, R. Jansky, J. Janssen, M. Janus, G. Jarlskog, N. Javadov, T. Javůrek, L. Jeanty, J. Jejelava, G.-Y. Jeng, D. Jennens, P. Jenni, J. Jentzsch, C. Jeske, S. Jézéquel, H. Ji, J. Jia, Y. Jiang, S. Jiggins, J. Jimenez Pena, S. Jin, A. Jinaru, O. Jinnouchi, M. D. Joergensen, P. Johansson, K. A. Johns, K. Jon-And, G. Jones, R. W. L. Jones, T. J. Jones, J. Jongmanns, P. M. Jorge, K. D. Joshi, J. Jovicevic, X. Ju, C. A. Jung, P. Jussel, A. Juste Rozas, M. Kaci, A. Kaczmarska, M. Kado, H. Kagan, M. Kagan, S. J. Kahn, E. Kajomovitz, C. W. Kalderon, S. Kama, A. Kamenshchikov, N. Kanaya, S. Kaneti, V. A. Kantserov, J. Kanzaki, B. Kaplan, L. S. Kaplan, A. Kapliy, D. Kar, K. Karakostas, A. Karamaoun, N. Karastathis, M. J. Kareem, M. Karnevskiy, S. N. Karpov, Z. M. Karpova, K. Karthik, V. Kartvelishvili, A. N. Karyukhin, L. Kashif, R. D. Kass, A. Kastanas, Y. Kataoka, A. Katre, J. Katzy, K. Kawagoe, T. Kawamoto, G. Kawamura, S. Kazama, V. F. Kazanin, M. Y. Kazarinov, R. Keeler, R. Kehoe, J. S. Keller, J. J. Kempster, H. Keoshkerian, O. Kepka, B. P. Kerševan, S. Kersten, R. A. Keyes, F. Khalil-zada, H. Khandanyan, A. Khanov, A. G. Kharlamov, T. J. Khoo, V. Khovanskiy, E. Khramov, J. Khubua, H. Y. Kim, H. Kim, S. H. Kim, Y. Kim, N. Kimura, O. M. Kind, B. T. King, M. King, S. B. King, J. Kirk, A. E. Kiryunin, T. Kishimoto, D. Kisielewska, F. Kiss, K. Kiuchi, O. Kivernyk, E. Kladiva, M. H. Klein, M. Klein, U. Klein, K. Kleinknecht, P. Klimek, A. Klimentov, R. Klingenberg, J. A. Klinger, T. Klioutchnikova, E.-E. Kluge, P. Kluit, S. Kluth, J. Knapik, E. Kneringer, E. B. F. G. Knoops, A. Knue, A. Kobayashi, D. Kobayashi, T. Kobayashi, M. Kobel, M. Kocian, P. Kodys, T. Koffas, E. Koffeman, L. A. Kogan, S. Kohlmann, Z. Kohout, T. Kohriki, T. Koi, H. Kolanoski, I. Koletsou, A. A. Komar, Y. Komori, T. Kondo, N. Kondrashova, K. Köneke, A. C. König, T. Kono, R. Konoplich, N. Konstantinidis, R. Kopeliansky, S. Koperny, L. Köpke, A. K. Kopp, K. Korcyl, K. Kordas, A. Korn, A. A. Korol, I. Korolkov, E. V. Korolkova, O. Kortner, S. Kortner, T. Kosek, V. V. Kostyukhin, V. M. Kotov, A. Kotwal, A. Kourkoumeli-Charalampidi, C. Kourkoumelis, V. Kouskoura, A. Koutsman, R. Kowalewski, T. Z. Kowalski, W. Kozanecki, A. S. Kozhin, V. A. Kramarenko, G. Kramberger, D. Krasnopevtsev, M. W. Krasny, A. Krasznahorkay, J. K. Kraus, A. Kravchenko, S. Kreiss, M. Kretz, J. Kretzschmar, K. Kreutzfeldt, P. Krieger, K. Krizka, K. Kroeninger, H. Kroha, J. Kroll, J. Kroseberg, J. Krstic, U. Kruchonak, H. Krüger, N. Krumnack, Z. V. Krumshteyn, A. Kruse, M. C. Kruse, M. Kruskal, T. Kubota, H. Kucuk, S. Kuday, S. Kuehn, A. Kugel, F. Kuger, A. Kuhl, T. Kuhl, V. Kukhtin, Y. Kulchitsky, S. Kuleshov, M. Kuna, T. Kunigo, A. Kupco, H. Kurashige, Y. A. Kurochkin, V. Kus, E. S. Kuwertz, M. Kuze, J. Kvita, T. Kwan, D. Kyriazopoulos, A. La Rosa, J. L. La Rosa Navarro, L. La Rotonda, C. Lacasta, F. Lacava, J. Lacey, H. Lacker, D. Lacour, V. R. Lacuesta, E. Ladygin, R. Lafaye, B. Laforge, T. Lagouri, S. Lai, L. Lambourne, S. Lammers, C. L. Lampen, W. Lampl, E. Lançon, U. Landgraf, M. P. J. Landon, V. S. Lang, J. C. Lange, A. J. Lankford, F. Lanni, K. Lantzsch, A. Lanza, S. Laplace, C. Lapoire, J. F. Laporte, T. Lari, F. Lasagni Manghi, M. Lassnig, P. Laurelli, W. Lavrijsen, A. T. Law, P. Laycock, T. Lazovich, O. Le Dortz, E. Le Guirriec, E. Le Menedeu, M. LeBlanc, T. LeCompte, F. Ledroit-Guillon, C. A. Lee, S. C. Lee, L. Lee, G. Lefebvre, M. Lefebvre, F. Legger, C. Leggett, A. Lehan, G. Lehmann Miotto, X. Lei, W. A. Leight, A. Leisos, A. G. Leister, M. A. L. Leite, R. Leitner, D. Lellouch, B. Lemmer, K. J. C. Leney, T. Lenz, B. Lenzi, R. Leone, S. Leone, C. Leonidopoulos, S. Leontsinis, C. Leroy, C. G. Lester, M. Levchenko, J. Levêque, D. Levin, L. J. Levinson, M. Levy, A. Lewis, A. M. Leyko, M. Leyton, B. Li, H. Li, H. L. Li, L. Li, L. Li, S. Li, Y. Li, Z. Liang, H. Liao, B. Liberti, A. Liblong, P. Lichard, K. Lie, J. Liebal, W. Liebig, C. Limbach, A. Limosani, S. C. Lin, T. H. Lin, F. Linde, B. E. Lindquist, J. T. Linnemann, E. Lipeles, A. Lipniacka, M. Lisovyi, T. M. Liss, D. Lissauer, A. Lister, A. M. Litke, B. Liu, D. Liu, H. Liu, J. Liu, J. B. Liu, K. Liu, L. Liu, M. Liu, M. Liu, Y. Liu, M. Livan, A. Lleres, J. Llorente Merino, S. L. Lloyd, F. Lo Sterzo, E. Lobodzinska, P. Loch, W. S. Lockman, F. K. Loebinger, A. E. Loevschall-Jensen, A. Loginov, T. Lohse, K. Lohwasser, M. Lokajicek, B. A. Long, J. D. Long, R. E. Long, K. A. Looper, L. Lopes, D. Lopez Mateos, B. Lopez Paredes, I. Lopez Paz, J. Lorenz, N. Lorenzo Martinez, M. Losada, P. Loscutoff, P. J. Lösel, X. Lou, A. Lounis, J. Love, P. A. Love, N. Lu, H. J. Lubatti, C. Luci, A. Lucotte, F. Luehring, W. Lukas, L. Luminari, O. Lundberg, B. Lund-Jensen, D. Lynn, R. Lysak, E. Lytken, H. Ma, L. L. Ma, G. Maccarrone, A. Macchiolo, C. M. Macdonald, J. Machado Miguens, D. Macina, D. Madaffari, R. Madar, H. J. Maddocks, W. F. Mader, A. Madsen, S. Maeland, T. Maeno, A. Maevskiy, E. Magradze, K. Mahboubi, J. Mahlstedt, C. Maiani, C. Maidantchik, A. A. Maier, T. Maier, A. Maio, S. Majewski, Y. Makida, N. Makovec, B. Malaescu, Pa. Malecki, V. P. Maleev, F. Malek, U. Mallik, D. Malon, C. Malone, S. Maltezos, V. M. Malyshev, S. Malyukov, J. Mamuzic, G. Mancini, B. Mandelli, L. Mandelli, I. Mandić, R. Mandrysch, J. Maneira, A. Manfredini, L. Manhaes de Andrade Filho, J. Manjarres Ramos, A. Mann, P. M. Manning, A. Manousakis-Katsikakis, B. Mansoulie, R. Mantifel, M. Mantoani, L. Mapelli, L. March, G. Marchiori, M. Marcisovsky, C. P. Marino, M. Marjanovic, D. E. Marley, F. Marroquim, S. P. Marsden, Z. Marshall, L. F. Marti, S. Marti-Garcia, B. Martin, T. A. Martin, V. J. Martin, B. Martin dit Latour, M. Martinez, S. Martin-Haugh, V. S. Martoiu, A. C. Martyniuk, M. Marx, F. Marzano, A. Marzin, L. Masetti, T. Mashimo, R. Mashinistov, J. Masik, A. L. Maslennikov, I. Massa, L. Massa, N. Massol, P. Mastrandrea, A. Mastroberardino, T. Masubuchi, P. Mättig, J. Mattmann, J. Maurer, S. J. Maxfield, D. A. Maximov, R. Mazini, S. M. Mazza, L. Mazzaferro, G. Mc Goldrick, S. P. Mc Kee, A. McCarn, R. L. McCarthy, T. G. McCarthy, N. A. McCubbin, K. W. McFarlane, J. A. Mcfayden, G. Mchedlidze, S. J. McMahon, R. A. McPherson, M. Medinnis, S. Meehan, S. Mehlhase, A. Mehta, K. Meier, C. Meineck, B. Meirose, B. R. Mellado Garcia, F. Meloni, A. Mengarelli, S. Menke, E. Meoni, K. M. Mercurio, S. Mergelmeyer, P. Mermod, L. Merola, C. Meroni, F. S. Merritt, A. Messina, J. Metcalfe, A. S. Mete, C. Meyer, C. Meyer, J-P. Meyer, J. Meyer, R. P. Middleton, S. Miglioranzi, L. Mijović, G. Mikenberg, M. Mikestikova, M. Mikuž, M. Milesi, A. Milic, D. W. Miller, C. Mills, A. Milov, D. A. Milstead, A. A. Minaenko, Y. Minami, I. A. Minashvili, A. I. Mincer, B. Mindur, M. Mineev, Y. Ming, L. M. Mir, T. Mitani, J. Mitrevski, V. A. Mitsou, A. Miucci, P. S. Miyagawa, J. U. Mjörnmark, T. Moa, K. Mochizuki, S. Mohapatra, W. Mohr, S. Molander, R. Moles-Valls, K. Mönig, C. Monini, J. Monk, E. Monnier, J. Montejo Berlingen, F. Monticelli, S. Monzani, R. W. Moore, N. Morange, D. Moreno, M. Moreno Llácer, P. Morettini, M. Morgenstern, M. Morii, M. Morinaga, V. Morisbak, S. Moritz, A. K. Morley, G. Mornacchi, J. D. Morris, S. S. Mortensen, A. Morton, L. Morvaj, M. Mosidze, J. Moss, K. Motohashi, R. Mount, E. Mountricha, S. V. Mouraviev, E. J. W. Moyse, S. Muanza, R. D. Mudd, F. Mueller, J. Mueller, R. S. P. Mueller, T. Mueller, D. Muenstermann, P. Mullen, G. A. Mullier, J. A. Murillo Quijada, W. J. Murray, H. Musheghyan, E. Musto, A. G. Myagkov, M. Myska, O. Nackenhorst, J. Nadal, K. Nagai, R. Nagai, Y. Nagai, K. Nagano, A. Nagarkar, Y. Nagasaka, K. Nagata, M. Nagel, E. Nagy, A. M. Nairz, Y. Nakahama, K. Nakamura, T. Nakamura, I. Nakano, H. Namasivayam, R. F. Naranjo Garcia, R. Narayan, T. Naumann, G. Navarro, R. Nayyar, H. A. Neal, P. Yu. Nechaeva, T. J. Neep, P. D. Nef, A. Negri, M. Negrini, S. Nektarijevic, C. Nellist, A. Nelson, S. Nemecek, P. Nemethy, A. A. Nepomuceno, M. Nessi, M. S. Neubauer, M. Neumann, R. M. Neves, P. Nevski, P. R. Newman, D. H. Nguyen, R. B. Nickerson, R. Nicolaidou, B. Nicquevert, J. Nielsen, N. Nikiforou, A. Nikiforov, V. Nikolaenko, I. Nikolic-Audit, K. Nikolopoulos, J. K. Nilsen, P. Nilsson, Y. Ninomiya, A. Nisati, R. Nisius, T. Nobe, M. Nomachi, I. Nomidis, T. Nooney, S. Norberg, M. Nordberg, O. Novgorodova, S. Nowak, M. Nozaki, L. Nozka, K. Ntekas, G. Nunes Hanninger, T. Nunnemann, E. Nurse, F. Nuti, B. J. O’Brien, F. O’grady, D. C. O’Neil, V. O’Shea, F. G. Oakham, H. Oberlack, T. Obermann, J. Ocariz, A. Ochi, I. Ochoa, J. P. Ochoa-Ricoux, S. Oda, S. Odaka, H. Ogren, A. Oh, S. H. Oh, C. C. Ohm, H. Ohman, H. Oide, W. Okamura, H. Okawa, Y. Okumura, T. Okuyama, A. Olariu, S. A. Olivares Pino, D. Oliveira Damazio, E. Oliver Garcia, A. Olszewski, J. Olszowska, A. Onofre, P. U. E. Onyisi, C. J. Oram, M. J. Oreglia, Y. Oren, D. Orestano, N. Orlando, C. Oropeza Barrera, R. S. Orr, B. Osculati, R. Ospanov, G. Otero y Garzon, H. Otono, M. Ouchrif, E. A. Ouellette, F. Ould-Saada, A. Ouraou, K. P. Oussoren, Q. Ouyang, A. Ovcharova, M. Owen, R. E. Owen, V. E. Ozcan, N. Ozturk, K. Pachal, A. Pacheco Pages, C. Padilla Aranda, M. Pagáčová, S. Pagan Griso, E. Paganis, F. Paige, P. Pais, K. Pajchel, G. Palacino, S. Palestini, M. Palka, D. Pallin, A. Palma, Y. B. Pan, E. Panagiotopoulou, C. E. Pandini, J. G. Panduro Vazquez, P. Pani, S. Panitkin, D. Pantea, L. Paolozzi, Th. D. Papadopoulou, K. Papageorgiou, A. Paramonov, D. Paredes Hernandez, M. A. Parker, K. A. Parker, F. Parodi, J. A. Parsons, U. Parzefall, E. Pasqualucci, S. Passaggio, F. Pastore, Fr. Pastore, G. Pásztor, S. Pataraia, N. D. Patel, J. R. Pater, T. Pauly, J. Pearce, B. Pearson, L. E. Pedersen, M. Pedersen, S. Pedraza Lopez, R. Pedro, S. V. Peleganchuk, D. Pelikan, O. Penc, C. Peng, H. Peng, B. Penning, J. Penwell, D. V. Perepelitsa, E. Perez Codina, M. T. Pérez García-Estañ, L. Perini, H. Pernegger, S. Perrella, R. Peschke, V. D. Peshekhonov, K. Peters, R. F. Y. Peters, B. A. Petersen, T. C. Petersen, E. Petit, A. Petridis, C. Petridou, E. Petrolo, F. Petrucci, N. E. Pettersson, R. Pezoa, P. W. Phillips, G. Piacquadio, E. Pianori, A. Picazio, E. Piccaro, M. Piccinini, M. A. Pickering, R. Piegaia, D. T. Pignotti, J. E. Pilcher, A. D. Pilkington, J. Pina, M. Pinamonti, J. L. Pinfold, A. Pingel, B. Pinto, S. Pires, H. Pirumov, M. Pitt, C. Pizio, L. Plazak, M.-A. Pleier, V. Pleskot, E. Plotnikova, P. Plucinski, D. Pluth, R. Poettgen, L. Poggioli, D. Pohl, G. Polesello, A. Poley, A. Policicchio, R. Polifka, A. Polini, C. S. Pollard, V. Polychronakos, K. Pommès, L. Pontecorvo, B. G. Pope, G. A. Popeneciu, D. S. Popovic, A. Poppleton, S. Pospisil, K. Potamianos, I. N. Potrap, C. J. Potter, C. T. Potter, G. Poulard, J. Poveda, V. Pozdnyakov, P. Pralavorio, A. Pranko, S. Prasad, S. Prell, D. Price, L. E. Price, M. Primavera, S. Prince, M. Proissl, K. Prokofiev, F. Prokoshin, E. Protopapadaki, S. Protopopescu, J. Proudfoot, M. Przybycien, E. Ptacek, D. Puddu, E. Pueschel, D. Puldon, M. Purohit, P. Puzo, J. Qian, G. Qin, Y. Qin, A. Quadt, D. R. Quarrie, W. B. Quayle, M. Queitsch-Maitland, D. Quilty, S. Raddum, V. Radeka, V. Radescu, S. K. Radhakrishnan, P. Radloff, P. Rados, F. Ragusa, G. Rahal, S. Rajagopalan, M. Rammensee, C. Rangel-Smith, F. Rauscher, S. Rave, T. Ravenscroft, M. Raymond, A. L. Read, N. P. Readioff, D. M. Rebuzzi, A. Redelbach, G. Redlinger, R. Reece, K. Reeves, L. Rehnisch, H. Reisin, M. Relich, C. Rembser, H. Ren, A. Renaud, M. Rescigno, S. Resconi, O. L. Rezanova, P. Reznicek, R. Rezvani, R. Richter, S. Richter, E. Richter-Was, O. Ricken, M. Ridel, P. Rieck, C. J. Riegel, J. Rieger, M. Rijssenbeek, A. Rimoldi, L. Rinaldi, B. Ristić, E. Ritsch, I. Riu, F. Rizatdinova, E. Rizvi, S. H. Robertson, A. Robichaud-Veronneau, D. Robinson, J. E. M. Robinson, A. Robson, C. Roda, S. Roe, O. Røhne, S. Rolli, A. Romaniouk, M. Romano, S. M. Romano Saez, E. Romero Adam, N. Rompotis, M. Ronzani, L. Roos, E. Ros, S. Rosati, K. Rosbach, P. Rose, P. L. Rosendahl, O. Rosenthal, V. Rossetti, E. Rossi, L. P. Rossi, R. Rosten, M. Rotaru, I. Roth, J. Rothberg, D. Rousseau, C. R. Royon, A. Rozanov, Y. Rozen, X. Ruan, F. Rubbo, I. Rubinskiy, V. I. Rud, C. Rudolph, M. S. Rudolph, F. Rühr, A. Ruiz-Martinez, Z. Rurikova, N. A. Rusakovich, A. Ruschke, H. L. Russell, J. P. Rutherfoord, N. Ruthmann, Y. F. Ryabov, M. Rybar, G. Rybkin, N. C. Ryder, A. F. Saavedra, G. Sabato, S. Sacerdoti, A. Saddique, H. F-W. Sadrozinski, R. Sadykov, F. Safai Tehrani, M. Saimpert, H. Sakamoto, Y. Sakurai, G. Salamanna, A. Salamon, M. Saleem, D. Salek, P. H. Sales De Bruin, D. Salihagic, A. Salnikov, J. Salt, D. Salvatore, F. Salvatore, A. Salvucci, A. Salzburger, D. Sampsonidis, A. Sanchez, J. Sánchez, V. Sanchez Martinez, H. Sandaker, R. L. Sandbach, H. G. Sander, M. P. Sanders, M. Sandhoff, C. Sandoval, R. Sandstroem, D. P. C. Sankey, M. Sannino, A. Sansoni, C. Santoni, R. Santonico, H. Santos, I. Santoyo Castillo, K. Sapp, A. Sapronov, J. G. Saraiva, B. Sarrazin, O. Sasaki, Y. Sasaki, K. Sato, G. Sauvage, E. Sauvan, G. Savage, P. Savard, C. Sawyer, L. Sawyer, J. Saxon, C. Sbarra, A. Sbrizzi, T. Scanlon, D. A. Scannicchio, M. Scarcella, V. Scarfone, J. Schaarschmidt, P. Schacht, D. Schaefer, R. Schaefer, J. Schaeffer, S. Schaepe, S. Schaetzel, U. Schäfer, A. C. Schaffer, D. Schaile, R. D. Schamberger, V. Scharf, V. A. Schegelsky, D. Scheirich, M. Schernau, C. Schiavi, C. Schillo, M. Schioppa, S. Schlenker, E. Schmidt, K. Schmieden, C. Schmitt, S. Schmitt, S. Schmitt, B. Schneider, Y. J. Schnellbach, U. Schnoor, L. Schoeffel, A. Schoening, B. D. Schoenrock, E. Schopf, A. L. S. Schorlemmer, M. Schott, D. Schouten, J. Schovancova, S. Schramm, M. Schreyer, C. Schroeder, N. Schuh, M. J. Schultens, H.-C. Schultz-Coulon, H. Schulz, M. Schumacher, B. A. Schumm, Ph. Schune, C. Schwanenberger, A. Schwartzman, T. A. Schwarz, Ph. Schwegler, H. Schweiger, Ph. Schwemling, R. Schwienhorst, J. Schwindling, T. Schwindt, F. G. Sciacca, E. Scifo, G. Sciolla, F. Scuri, F. Scutti, J. Searcy, G. Sedov, E. Sedykh, P. Seema, S. C. Seidel, A. Seiden, F. Seifert, J. M. Seixas, G. Sekhniaidze, K. Sekhon, S. J. Sekula, D. M. Seliverstov, N. Semprini-Cesari, C. Serfon, L. Serin, L. Serkin, T. Serre, M. Sessa, R. Seuster, H. Severini, T. Sfiligoj, F. Sforza, A. Sfyrla, E. Shabalina, M. Shamim, L. Y. Shan, R. Shang, J. T. Shank, M. Shapiro, P. B. Shatalov, K. Shaw, S. M. Shaw, A. Shcherbakova, C. Y. Shehu, P. Sherwood, L. Shi, S. Shimizu, C. O. Shimmin, M. Shimojima, M. Shiyakova, A. Shmeleva, D. Shoaleh Saadi, M. J. Shochet, S. Shojaii, S. Shrestha, E. Shulga, M. A. Shupe, S. Shushkevich, P. Sicho, P. E. Sidebo, O. Sidiropoulou, D. Sidorov, A. Sidoti, F. Siegert, Dj. Sijacki, J. Silva, Y. Silver, S. B. Silverstein, V. Simak, O. Simard, Lj. Simic, S. Simion, E. Simioni, B. Simmons, D. Simon, R. Simoniello, P. Sinervo, N. B. Sinev, M. Sioli, G. Siragusa, A. N. Sisakyan, S. Yu. Sivoklokov, J. Sjölin, T. B. Sjursen, M. B. Skinner, H. P. Skottowe, P. Skubic, M. Slater, T. Slavicek, M. Slawinska, K. Sliwa, V. Smakhtin, B. H. Smart, L. Smestad, S. Yu. Smirnov, Y. Smirnov, L. N. Smirnova, O. Smirnova, M. N. K. Smith, R. W. Smith, M. Smizanska, K. Smolek, A. A. Snesarev, G. Snidero, S. Snyder, R. Sobie, F. Socher, A. Soffer, D. A. Soh, C. A. Solans, M. Solar, J. Solc, E. Yu. Soldatov, U. Soldevila, A. A. Solodkov, A. Soloshenko, O. V. Solovyanov, V. Solovyev, P. Sommer, H. Y. Song, N. Soni, A. Sood, A. Sopczak, B. Sopko, V. Sopko, V. Sorin, D. Sosa, M. Sosebee, C. L. Sotiropoulou, R. Soualah, A. M. Soukharev, D. South, B. C. Sowden, S. Spagnolo, M. Spalla, F. Spanò, W. R. Spearman, D. Sperlich, F. Spettel, R. Spighi, G. Spigo, L. A. Spiller, M. Spousta, T. Spreitzer, R. D. St. Denis, S. Staerz, J. Stahlman, R. Stamen, S. Stamm, E. Stanecka, C. Stanescu, M. Stanescu-Bellu, M. M. Stanitzki, S. Stapnes, E. A. Starchenko, J. Stark, P. Staroba, P. Starovoitov, R. Staszewski, P. Stavina, P. Steinberg, B. Stelzer, H. J. Stelzer, O. Stelzer-Chilton, H. Stenzel, G. A. Stewart, J. A. Stillings, M. C. Stockton, M. Stoebe, G. Stoicea, P. Stolte, S. Stonjek, A. R. Stradling, A. Straessner, M. E. Stramaglia, J. Strandberg, S. Strandberg, A. Strandlie, E. Strauss, M. Strauss, P. Strizenec, R. Ströhmer, D. M. Strom, R. Stroynowski, A. Strubig, S. A. Stucci, B. Stugu, N. A. Styles, D. Su, J. Su, R. Subramaniam, A. Succurro, Y. Sugaya, C. Suhr, M. Suk, V. V. Sulin, S. Sultansoy, T. Sumida, S. Sun, X. Sun, J. E. Sundermann, K. Suruliz, G. Susinno, M. R. Sutton, S. Suzuki, M. Svatos, S. Swedish, M. Swiatlowski, I. Sykora, T. Sykora, D. Ta, C. Taccini, K. Tackmann, J. Taenzer, A. Taffard, R. Tafirout, N. Taiblum, H. Takai, R. Takashima, H. Takeda, T. Takeshita, Y. Takubo, M. Talby, A. A. Talyshev, J. Y. C. Tam, K. G. Tan, J. Tanaka, R. Tanaka, S. Tanaka, B. B. Tannenwald, N. Tannoury, S. Tapprogge, S. Tarem, F. Tarrade, G. F. Tartarelli, P. Tas, M. Tasevsky, T. Tashiro, E. Tassi, A. Tavares Delgado, Y. Tayalati, F. E. Taylor, G. N. Taylor, W. Taylor, F. A. Teischinger, M. Teixeira Dias Castanheira, P. Teixeira-Dias, K. K. Temming, H. Ten Kate, P. K. Teng, J. J. Teoh, F. Tepel, S. Terada, K. Terashi, J. Terron, S. Terzo, M. Testa, R. J. Teuscher, T. Theveneaux-Pelzer, J. P. Thomas, J. Thomas-Wilsker, E. N. Thompson, P. D. Thompson, R. J. Thompson, A. S. Thompson, L. A. Thomsen, E. Thomson, M. Thomson, R. P. Thun, M. J. Tibbetts, R. E. Ticse Torres, V. O. Tikhomirov, Yu. A. Tikhonov, S. Timoshenko, E. Tiouchichine, P. Tipton, S. Tisserant, K. Todome, T. Todorov, S. Todorova-Nova, J. Tojo, S. Tokár, K. Tokushuku, K. Tollefson, E. Tolley, L. Tomlinson, M. Tomoto, L. Tompkins, K. Toms, E. Torrence, H. Torres, E. Torró Pastor, J. Toth, F. Touchard, D. R. Tovey, T. Trefzger, L. Tremblet, A. Tricoli, I. M. Trigger, S. Trincaz-Duvoid, M. F. Tripiana, W. Trischuk, B. Trocmé, C. Troncon, M. Trottier-McDonald, M. Trovatelli, P. True, L. Truong, M. Trzebinski, A. Trzupek, C. Tsarouchas, J. C-L. Tseng, P. V. Tsiareshka, D. Tsionou, G. Tsipolitis, N. Tsirintanis, S. Tsiskaridze, V. Tsiskaridze, E. G. Tskhadadze, I. I. Tsukerman, V. Tsulaia, S. Tsuno, D. Tsybychev, A. Tudorache, V. Tudorache, A. N. Tuna, S. A. Tupputi, S. Turchikhin, D. Turecek, R. Turra, A. J. Turvey, P. M. Tuts, A. Tykhonov, M. Tylmad, M. Tyndel, I. Ueda, R. Ueno, M. Ughetto, M. Ugland, M. Uhlenbrock, F. Ukegawa, G. Unal, A. Undrus, G. Unel, F. C. Ungaro, Y. Unno, C. Unverdorben, J. Urban, P. Urquijo, P. Urrejola, G. Usai, A. Usanova, L. Vacavant, V. Vacek, B. Vachon, C. Valderanis, N. Valencic, S. Valentinetti, A. Valero, L. Valery, S. Valkar, E. Valladolid Gallego, S. Vallecorsa, J. A. Valls Ferrer, W. Van Den Wollenberg, P. C. Van Der Deijl, R. van der Geer, H. van der Graaf, R. Van Der Leeuw, N. van Eldik, P. van Gemmeren, J. Van Nieuwkoop, I. van Vulpen, M. C. van Woerden, M. Vanadia, W. Vandelli, R. Vanguri, A. Vaniachine, F. Vannucci, G. Vardanyan, R. Vari, E. W. Varnes, T. Varol, D. Varouchas, A. Vartapetian, K. E. Varvell, F. Vazeille, T. Vazquez Schroeder, J. Veatch, L. M. Veloce, F. Veloso, T. Velz, S. Veneziano, A. Ventura, D. Ventura, M. Venturi, N. Venturi, A. Venturini, V. Vercesi, M. Verducci, W. Verkerke, J. C. Vermeulen, A. Vest, M. C. Vetterli, O. Viazlo, I. Vichou, T. Vickey, O. E. Vickey Boeriu, G. H. A. Viehhauser, S. Viel, R. Vigne, M. Villa, M. Villaplana Perez, E. Vilucchi, M. G. Vincter, V. B. Vinogradov, I. Vivarelli, F. Vives Vaque, S. Vlachos, D. Vladoiu, M. Vlasak, M. Vogel, P. Vokac, G. Volpi, M. Volpi, H. von der Schmitt, H. von Radziewski, E. von Toerne, V. Vorobel, K. Vorobev, M. Vos, R. Voss, J. H. Vossebeld, N. Vranjes, M. Vranjes Milosavljevic, V. Vrba, M. Vreeswijk, R. Vuillermet, I. Vukotic, Z. Vykydal, P. Wagner, W. Wagner, H. Wahlberg, S. Wahrmund, J. Wakabayashi, J. Walder, R. Walker, W. Walkowiak, C. Wang, F. Wang, H. Wang, H. Wang, J. Wang, J. Wang, K. Wang, R. Wang, S. M. Wang, T. Wang, T. Wang, X. Wang, C. Wanotayaroj, A. Warburton, C. P. Ward, D. R. Wardrope, M. Warsinsky, A. Washbrook, C. Wasicki, P. M. Watkins, A. T. Watson, I. J. Watson, M. F. Watson, G. Watts, S. Watts, B. M. Waugh, S. Webb, M. S. Weber, S. W. Weber, J. S. Webster, A. R. Weidberg, B. Weinert, J. Weingarten, C. Weiser, H. Weits, P. S. Wells, T. Wenaus, T. Wengler, S. Wenig, N. Wermes, M. Werner, P. Werner, M. Wessels, J. Wetter, K. Whalen, A. M. Wharton, A. White, M. J. White, R. White, S. White, D. Whiteson, F. J. Wickens, W. Wiedenmann, M. Wielers, P. Wienemann, C. Wiglesworth, L. A. M. Wiik-Fuchs, A. Wildauer, H. G. Wilkens, H. H. Williams, S. Williams, C. Willis, S. Willocq, A. Wilson, J. A. Wilson, I. Wingerter-Seez, F. Winklmeier, B. T. Winter, M. Wittgen, J. Wittkowski, S. J. Wollstadt, M. W. Wolter, H. Wolters, B. K. Wosiek, J. Wotschack, M. J. Woudstra, K. W. Wozniak, M. Wu, M. Wu, S. L. Wu, X. Wu, Y. Wu, T. R. Wyatt, B. M. Wynne, S. Xella, D. Xu, L. Xu, B. Yabsley, S. Yacoob, R. Yakabe, M. Yamada, Y. Yamaguchi, A. Yamamoto, S. Yamamoto, T. Yamanaka, K. Yamauchi, Y. Yamazaki, Z. Yan, H. Yang, H. Yang, Y. Yang, W-M. Yao, Y. Yasu, E. Yatsenko, K. H. Yau Wong, J. Ye, S. Ye, I. Yeletskikh, A. L. Yen, E. Yildirim, K. Yorita, R. Yoshida, K. Yoshihara, C. Young, C. J. S. Young, S. Youssef, D. R. Yu, J. Yu, J. M. Yu, J. Yu, L. Yuan, A. Yurkewicz, I. Yusuff, B. Zabinski, R. Zaidan, A. M. Zaitsev, J. Zalieckas, A. Zaman, S. Zambito, L. Zanello, D. Zanzi, C. Zeitnitz, M. Zeman, A. Zemla, K. Zengel, O. Zenin, T. Ženiš, D. Zerwas, D. Zhang, F. Zhang, H. Zhang, J. Zhang, L. Zhang, R. Zhang, X. Zhang, Z. Zhang, X. Zhao, Y. Zhao, Z. Zhao, A. Zhemchugov, J. Zhong, B. Zhou, C. Zhou, L. Zhou, L. Zhou, N. Zhou, C. G. Zhu, H. Zhu, J. Zhu, Y. Zhu, X. Zhuang, K. Zhukov, A. Zibell, D. Zieminska, N. I. Zimine, C. Zimmermann, S. Zimmermann, Z. Zinonos, M. Zinser, M. Ziolkowski, L. Živković, G. Zobernig, A. Zoccoli, M. zur Nedden, G. Zurzolo, L. Zwalinski, ATLAS Collaboration

**Affiliations:** Department of Physics, University of Adelaide, Adelaide, Australia; Physics Department, SUNY Albany, Albany, NY USA; Department of Physics, University of Alberta, Edmonton, AB Canada; Department of Physics, Ankara University, Ankara, Turkey; Istanbul Aydin University, Istanbul, Turkey; Division of Physics, TOBB University of Economics and Technology, Ankara, Turkey; LAPP, CNRS/IN2P3 and Université Savoie Mont Blanc, Annecy-le-Vieux, France; High Energy Physics Division, Argonne National Laboratory, Argonne, IL USA; Department of Physics, University of Arizona, Tucson, AZ USA; Department of Physics, The University of Texas at Arlington, Arlington, TX USA; Physics Department, University of Athens, Athens, Greece; Physics Department, National Technical University of Athens, Zografou, Greece; Institute of Physics, Azerbaijan Academy of Sciences, Baku, Azerbaijan; Institut de Física d’Altes Energies and Departament de Física de la Universitat Autònoma de Barcelona, Barcelona, Spain; Institute of Physics, University of Belgrade, Belgrade, Serbia; Department for Physics and Technology, University of Bergen, Bergen, Norway; Physics Division, Lawrence Berkeley National Laboratory and University of California, Berkeley, CA USA; Department of Physics, Humboldt University, Berlin, Germany; Albert Einstein Center for Fundamental Physics and Laboratory for High Energy Physics, University of Bern, Bern, Switzerland; School of Physics and Astronomy, University of Birmingham, Birmingham, UK; Department of Physics, Bogazici University, Istanbul, Turkey; Department of Physics Engineering, Gaziantep University, Gaziantep, Turkey; Department of Physics, Dogus University, Gaziantep, Turkey; INFN Sezione di Bologna, Bologna, Italy; Dipartimento di Fisica e Astronomia, Università di Bologna, Bologna, Italy; Physikalisches Institut, University of Bonn, Bonn, Germany; Department of Physics, Boston University, Boston, MA USA; Department of Physics, Brandeis University, Waltham, MA USA; Universidade Federal do Rio De Janeiro COPPE/EE/IF, Rio de Janeiro, Brazil; Electrical Circuits Department, Federal University of Juiz de Fora (UFJF), Juiz de Fora, Brazil; Federal University of Sao Joao del Rei (UFSJ), Sao Joao del Rei, Brazil; Instituto de Fisica, Universidade de Sao Paulo, São Paulo, Brazil; Physics Department, Brookhaven National Laboratory, Upton, NY USA; National Institute of Physics and Nuclear Engineering, Bucharest, Romania; Physics Department, National Institute for Research and Development of Isotopic and Molecular Technologies, Cluj Napoca, Romania; University Politehnica Bucharest, Bucharest, Romania; West University in Timisoara, Timisoara, Romania; Departamento de Física, Universidad de Buenos Aires, Buenos Aires, Argentina; Cavendish Laboratory, University of Cambridge, Cambridge, UK; Department of Physics, Carleton University, Ottawa, ON Canada; CERN, Geneva, Switzerland; Enrico Fermi Institute, University of Chicago, Chicago, IL USA; Departamento de Física, Pontificia Universidad Católica de Chile, Santiago, Chile; Departamento de Física, Universidad Técnica Federico Santa María, Valparaiso, Chile; Institute of High Energy Physics, Chinese Academy of Sciences, Beijing, China; Department of Modern Physics, University of Science and Technology of China, Hefei, Anhui China; Department of Physics, Nanjing University, Nanjing, Jiangsu China; School of Physics, Shandong University, Shandong, China; Shanghai Key Laboratory for Particle Physics and Cosmology, Department of Physics and Astronomy, Shanghai Jiao Tong University, Shanghai, China; Physics Department, Tsinghua University, Beijing, 100084 China; Laboratoire de Physique Corpusculaire, Clermont Université and Université Blaise Pascal and CNRS/IN2P3, Clermont-Ferrand, France; Nevis Laboratory, Columbia University, Irvington, NY USA; Niels Bohr Institute, University of Copenhagen, Copenhagen, Denmark; INFN Gruppo Collegato di Cosenza, Laboratori Nazionali di Frascati, Frascati, Italy; Dipartimento di Fisica, Università della Calabria, Rende, Italy; AGH University of Science and Technology, Faculty of Physics and Applied Computer Science, Kraków, Poland; Marian Smoluchowski Institute of Physics, Jagiellonian University, Kraków, Poland; Institute of Nuclear Physics, Polish Academy of Sciences, Kraków, Poland; Physics Department, Southern Methodist University, Dallas, TX USA; Physics Department, University of Texas at Dallas, Richardson, TX USA; DESY, Hamburg and Zeuthen, Germany; Institut für Experimentelle Physik IV, Technische Universität Dortmund, Dortmund, Germany; Institut für Kern- und Teilchenphysik, Technische Universität Dresden, Dresden, Germany; Department of Physics, Duke University, Durham, NC USA; SUPA-School of Physics and Astronomy, University of Edinburgh, Edinburgh, UK; INFN Laboratori Nazionali di Frascati, Frascati, Italy; Fakultät für Mathematik und Physik, Albert-Ludwigs-Universität, Freiburg, Germany; Section de Physique, Université de Genève, Geneva, Switzerland; INFN Sezione di Genova, Genoa, Italy; Dipartimento di Fisica, Università di Genova, Genoa, Italy; E. Andronikashvili Institute of Physics, Iv. Javakhishvili Tbilisi State University, Tbilisi, Georgia; High Energy Physics Institute, Tbilisi State University, Tbilisi, Georgia; II Physikalisches Institut, Justus-Liebig-Universität Giessen, Giessen, Germany; SUPA-School of Physics and Astronomy, University of Glasgow, Glasgow, UK; II Physikalisches Institut, Georg-August-Universität, Göttingen, Germany; Laboratoire de Physique Subatomique et de Cosmologie, Université Grenoble-Alpes, CNRS/IN2P3, Grenoble, France; Department of Physics, Hampton University, Hampton, VA USA; Laboratory for Particle Physics and Cosmology, Harvard University, Cambridge, MA USA; Kirchhoff-Institut für Physik, Ruprecht-Karls-Universität Heidelberg, Heidelberg, Germany; Physikalisches Institut, Ruprecht-Karls-Universität Heidelberg, Heidelberg, Germany; ZITI Institut für technische Informatik, Ruprecht-Karls-Universität Heidelberg, Mannheim, Germany; Faculty of Applied Information Science, Hiroshima Institute of Technology, Hiroshima, Japan; Department of Physics, The Chinese University of Hong Kong, Shatin, NT Hong Kong; Department of Physics, The University of Hong Kong, Hong Kong, Hong Kong; Department of Physics, The Hong Kong University of Science and Technology, Clear Water Bay, Kowloon, Hong Kong, China; Department of Physics, Indiana University, Bloomington, IN USA; Institut für Astro- und Teilchenphysik, Leopold-Franzens-Universität, Innsbruck, Austria; University of Iowa, Iowa City, IA USA; Department of Physics and Astronomy, Iowa State University, Ames, IA USA; Joint Institute for Nuclear Research, JINR Dubna, Dubna, Russia; KEK, High Energy Accelerator Research Organization, Tsukuba, Japan; Graduate School of Science, Kobe University, Kobe, Japan; Faculty of Science, Kyoto University, Kyoto, Japan; Kyoto University of Education, Kyoto, Japan; Department of Physics, Kyushu University, Fukuoka, Japan; Instituto de Física La Plata, Universidad Nacional de La Plata and CONICET, La Plata, Argentina; Physics Department, Lancaster University, Lancaster, UK; INFN Sezione di Lecce, Lecce, Italy; Dipartimento di Matematica e Fisica, Università del Salento, Lecce, Italy; Oliver Lodge Laboratory, University of Liverpool, Liverpool, UK; Department of Physics, Jožef Stefan Institute and University of Ljubljana, Ljubljana, Slovenia; School of Physics and Astronomy, Queen Mary University of London, London, UK; Department of Physics, Royal Holloway University of London, Surrey, UK; Department of Physics and Astronomy, University College London, London, UK; Louisiana Tech University, Ruston, LA USA; Laboratoire de Physique Nucléaire et de Hautes Energies, UPMC and Université Paris-Diderot and CNRS/IN2P3, Paris, France; Fysiska institutionen, Lunds universitet, Lund, Sweden; Departamento de Fisica Teorica C-15, Universidad Autonoma de Madrid, Madrid, Spain; Institut für Physik, Universität Mainz, Mainz, Germany; School of Physics and Astronomy, University of Manchester, Manchester, UK; CPPM, Aix-Marseille Université and CNRS/IN2P3, Marseille, France; Department of Physics, University of Massachusetts, Amherst, MA USA; Department of Physics, McGill University, Montreal, QC Canada; School of Physics, University of Melbourne, Melbourne, VIC Australia; Department of Physics, The University of Michigan, Ann Arbor, MI USA; Department of Physics and Astronomy, Michigan State University, East Lansing, MI USA; INFN Sezione di Milano, Milan, Italy; Dipartimento di Fisica, Università di Milano, Milan, Italy; B.I. Stepanov Institute of Physics, National Academy of Sciences of Belarus, Minsk, Republic of Belarus; National Scientific and Educational Centre for Particle and High Energy Physics, Minsk, Republic of Belarus; Department of Physics, Massachusetts Institute of Technology, Cambridge, MA USA; Group of Particle Physics, University of Montreal, Montreal, QC Canada; P.N. Lebedev Institute of Physics, Academy of Sciences, Moscow, Russia; Institute for Theoretical and Experimental Physics (ITEP), Moscow, Russia; National Research Nuclear University MEPhI, Moscow, Russia; D.V. Skobeltsyn Institute of Nuclear Physics, M.V. Lomonosov Moscow State University, Moscow, Russia; Fakultät für Physik, Ludwig-Maximilians-Universität München, Munich, Germany; Max-Planck-Institut für Physik (Werner-Heisenberg-Institut), Munich, Germany; Nagasaki Institute of Applied Science, Nagasaki, Japan; Graduate School of Science and Kobayashi-Maskawa Institute, Nagoya University, Nagoya, Japan; INFN Sezione di Napoli, Naples, Italy; Dipartimento di Fisica, Università di Napoli, Naples, Italy; Department of Physics and Astronomy, University of New Mexico, Albuquerque, NM USA; Institute for Mathematics, Astrophysics and Particle Physics, Radboud University Nijmegen/Nikhef, Nijmegen, The Netherlands; Nikhef National Institute for Subatomic Physics and University of Amsterdam, Amsterdam, The Netherlands; Department of Physics, Northern Illinois University, De Kalb, IL USA; Budker Institute of Nuclear Physics, SB RAS, Novosibirsk, Russia; Department of Physics, New York University, New York, NY USA; Ohio State University, Columbus, OH USA; Faculty of Science, Okayama University, Okayama, Japan; Homer L. Dodge Department of Physics and Astronomy, University of Oklahoma, Norman, OK USA; Department of Physics, Oklahoma State University, Stillwater, OK USA; Palacký University, RCPTM, Olomouc, Czech Republic; Center for High Energy Physics, University of Oregon, Eugene, OR USA; LAL, Université Paris-Sud and CNRS/IN2P3, Orsay, France; Graduate School of Science, Osaka University, Osaka, Japan; Department of Physics, University of Oslo, Oslo, Norway; Department of Physics, Oxford University, Oxford, UK; INFN Sezione di Pavia, Pavia, Italy; Dipartimento di Fisica, Università di Pavia, Pavia, Italy; Department of Physics, University of Pennsylvania, Philadelphia, PA USA; National Research Centre “Kurchatov Institute” B.P.Konstantinov, Petersburg Nuclear Physics Institute, St. Petersburg, Russia; INFN Sezione di Pisa, Pisa, Italy; Dipartimento di Fisica E. Fermi, Università di Pisa, Pisa, Italy; Department of Physics and Astronomy, University of Pittsburgh, Pittsburgh, PA USA; Laboratório de Instrumentação e Física Experimental de Partículas-LIP, Lisbon, Portugal; Faculdade de Ciências, Universidade de Lisboa, Lisbon, Portugal; Department of Physics, University of Coimbra, Coimbra, Portugal; Centro de Física Nuclear da Universidade de Lisboa, Lisbon, Portugal; Departamento de Fisica, Universidade do Minho, Braga, Portugal; Departamento de Fisica Teorica y del Cosmos and CAFPE, Universidad de Granada, Granada, Spain; Dep Fisica and CEFITEC of Faculdade de Ciencias e Tecnologia, Universidade Nova de Lisboa, Caparica, Portugal; Institute of Physics, Academy of Sciences of the Czech Republic, Prague, Czech Republic; Czech Technical University in Prague, Prague, Czech Republic; Faculty of Mathematics and Physics, Charles University in Prague, Prague, Czech Republic; State Research Center Institute for High Energy Physics, Protvino, Russia; Particle Physics Department, Rutherford Appleton Laboratory, Didcot, UK; INFN Sezione di Roma, Rome, Italy; Dipartimento di Fisica, Sapienza Università di Roma, Rome, Italy; INFN Sezione di Roma Tor Vergata, Rome, Italy; Dipartimento di Fisica, Università di Roma Tor Vergata, Rome, Italy; INFN Sezione di Roma Tre, Rome, Italy; Dipartimento di Matematica e Fisica, Università Roma Tre, Rome, Italy; Faculté des Sciences Ain Chock, Réseau Universitaire de Physique des Hautes Energies-Université Hassan II, Casablanca, Morocco; Centre National de l’Energie des Sciences Techniques Nucleaires, Rabat, Morocco; Faculté des Sciences Semlalia, Université Cadi Ayyad, LPHEA-Marrakech, Marrakech, Morocco; Faculté des Sciences, Université Mohamed Premier and LPTPM, Oujda, Morocco; Faculté des Sciences, Université Mohammed V-Agdal, Rabat, Morocco; DSM/IRFU (Institut de Recherches sur les Lois Fondamentales de l’Univers), CEA Saclay (Commissariat à l’Energie Atomique et aux Energies Alternatives), Gif-sur-Yvette, France; Santa Cruz Institute for Particle Physics, University of California Santa Cruz, Santa Cruz, CA USA; Department of Physics, University of Washington, Seattle, WA USA; Department of Physics and Astronomy, University of Sheffield, Sheffield, UK; Department of Physics, Shinshu University, Nagano, Japan; Fachbereich Physik, Universität Siegen, Siegen, Germany; Department of Physics, Simon Fraser University, Burnaby, BC Canada; SLAC National Accelerator Laboratory, Stanford, CA USA; Faculty of Mathematics, Physics and Informatics, Comenius University, Bratislava, Slovak Republic; Department of Subnuclear Physics, Institute of Experimental Physics of the Slovak Academy of Sciences, Kosice, Slovak Republic; Department of Physics, University of Cape Town, Cape Town, South Africa; Department of Physics, University of Johannesburg, Johannesburg, South Africa; School of Physics, University of the Witwatersrand, Johannesburg, South Africa; Department of Physics, Stockholm University, Stockholm, Sweden; The Oskar Klein Centre, Stockholm, Sweden; Physics Department, Royal Institute of Technology, Stockholm, Sweden; Departments of Physics and Astronomy and Chemistry, Stony Brook University, Stony Brook, NY USA; Department of Physics and Astronomy, University of Sussex, Brighton, UK; School of Physics, University of Sydney, Sydney, Australia; Institute of Physics, Academia Sinica, Taipei, Taiwan; Department of Physics, Technion: Israel Institute of Technology, Haifa, Israel; Raymond and Beverly Sackler School of Physics and Astronomy, Tel Aviv University, Tel Aviv, Israel; Department of Physics, Aristotle University of Thessaloniki, Thessaloníki, Greece; International Center for Elementary Particle Physics and Department of Physics, The University of Tokyo, Tokyo, Japan; Graduate School of Science and Technology, Tokyo Metropolitan University, Tokyo, Japan; Department of Physics, Tokyo Institute of Technology, Tokyo, Japan; Department of Physics, University of Toronto, Toronto, ON Canada; TRIUMF, Vancouver, BC Canada; Department of Physics and Astronomy, York University, Toronto, ON Canada; Faculty of Pure and Applied Sciences, University of Tsukuba, Tsukuba, Japan; Department of Physics and Astronomy, Tufts University, Medford, MA USA; Centro de Investigaciones, Universidad Antonio Narino, Bogotá, Colombia; Department of Physics and Astronomy, University of California Irvine, Irvine, CA USA; INFN Gruppo Collegato di Udine, Sezione di Trieste, Udine, Italy; ICTP, Trieste, Italy; Dipartimento di Chimica Fisica e Ambiente, Università di Udine, Udine, Italy; Department of Physics, University of Illinois, Urbana, IL USA; Department of Physics and Astronomy, University of Uppsala, Uppsala, Sweden; Instituto de Física Corpuscular (IFIC) and Departamento de Física Atómica, Molecular y Nuclear and Departamento de Ingeniería Electrónica and Instituto de Microelectrónica de Barcelona (IMB-CNM), University of Valencia and CSIC, Valencia, Spain; Department of Physics, University of British Columbia, Vancouver, BC Canada; Department of Physics and Astronomy, University of Victoria, Victoria, BC Canada; Department of Physics, University of Warwick, Coventry, UK; Waseda University, Tokyo, Japan; Department of Particle Physics, The Weizmann Institute of Science, Rehovot, Israel; Department of Physics, University of Wisconsin, Madison, WI USA; Fakultät für Physik und Astronomie, Julius-Maximilians-Universität, Würzburg, Germany; Fachbereich C Physik, Bergische Universität Wuppertal, Wuppertal, Germany; Department of Physics, Yale University, New Haven, CT USA; Yerevan Physics Institute, Yerevan, Armenia; Centre de Calcul de l’Institut National de Physique Nucléaire et de Physique des Particules (IN2P3), Villeurbanne, France; CERN, Geneva, Switzerland

## Abstract

Studies of the spin, parity and tensor couplings of the Higgs boson in the $H \rightarrow ZZ^{*} \rightarrow 4 \ell$, $H \rightarrow WW^{*} \rightarrow e \nu \mu \nu$ and $H \rightarrow \gamma \gamma$ decay processes at the LHC are presented. The investigations are based on $25\;\mathrm{fb}^{-1}$ of *pp* collision data collected by the ATLAS experiment at $\sqrt{s}=7$ TeV and $\sqrt{s}=8$ TeV. The Standard Model (SM) Higgs boson hypothesis, corresponding to the quantum numbers $J^{P}=0^{+}$, is tested against several alternative spin scenarios, including non-SM spin-0 and spin-2 models with universal and non-universal couplings to fermions and vector bosons. All tested alternative models are excluded in favour of the SM Higgs boson hypothesis at more than 99.9 % confidence level. Using the $H \rightarrow ZZ^{*} \rightarrow 4 \ell$ and $H \rightarrow WW^{*} \rightarrow e \nu \mu \nu$ decays, the tensor structure of the interaction between the spin-0 boson and the SM vector bosons is also investigated. The observed distributions of variables sensitive to the non-SM tensor couplings are compatible with the SM predictions and constraints on the non-SM couplings are derived.

## Introduction

The discovery of a Higgs boson by the ATLAS [1] and CMS [2] experiments at the Large Hadron Collider (LHC) at CERN marked the beginning of a new era of experimental studies of the properties of this new particle. In the Standard Model (SM), the Higgs boson is a CP-even scalar particle, $J^{CP}=0^{++}$.^1^ Theories of physics beyond the SM (BSM) often require an extended Higgs sector featuring several neutral Higgs bosons. Such cases may include CP-mixing in the Higgs boson interactions, which could result in observable differences in the kinematics of final-state particles produced in their decays. A review of the phenomenology in the determination of Higgs boson spin and CP properties can be found in Ref. [3] and references therein.

Previous determinations of the Higgs boson spin and CP quantum numbers by the ATLAS and CMS Collaborations are reported in Refs. [4, 5]. Results on the same subject have also been published by the D0 and CDF Collaborations in Ref. [6]. All these studies indicate the compatibility of the spin and CP properties of the observed Higgs boson with the SM predictions. The ATLAS measurement excluded several alternative spin and parity hypotheses in favour of the quantum numbers predicted by the SM. In addition to the exclusion of several non-SM spin hypotheses, the CMS measurement probed the tensor structure of the Higgs boson decay to SM vector bosons in the spin-0 scenario. This paper complements the previous ATLAS study of the Higgs boson spin and parity. The new study takes advantage of improvements to the analysis strategy and to the modelling used to describe alternative spin hypotheses, and includes studies on CP-mixing for the spin-0 scenario. The improved theoretical framework is based on the Higgs boson characterisation model described in Refs. [3, 7].

The study of the spin and parity properties of the Higgs boson presented in this paper is based on the $H \rightarrow \gamma \gamma$, $H \rightarrow ZZ^{*} \rightarrow 4 \ell$ and $H \rightarrow WW^{*} \rightarrow e \nu \mu \nu$ decay channels and their combination. The $H \rightarrow WW^{*} \rightarrow e \nu \mu \nu$ analysis is described in detail in a separate publication [8]. These analyses are based on 4.5 and 20.3 fb$^{-1}$ of *pp* collision data collected by the ATLAS experiment at centre-of-mass energies of 7 and 8 TeV, respectively. For the $H \rightarrow WW^{*} \rightarrow e \nu \mu \nu$ studies only the data collected at a centre-of-mass energy of 8 TeV are used. The SM hypothesis $J^P=0^+$ is compared to alternative spin-0 models: a pseudoscalar boson $J^P=0^-$ and a BSM scalar boson $J^P=0^+_h$ [9, 10], which describes the interaction of the Higgs boson with the SM vector bosons with higher-dimension operators discussed in Sect. 3.1. Graviton-like tensor models with $J^P=2^+$ with universal and non-universal couplings [3, 7] are also considered. In these tests of fixed spin and parity hypotheses it is assumed that the resonance decay involves only one CP eigenstate.

In addition to the fixed spin and parity hypothesis tests, the possible presence of BSM terms in the Lagrangian describing the *HVV* vertex^2^ of the spin-0 resonance is also investigated. The *HVV* interaction is described in terms of an effective Lagrangian that contains the SM interaction and BSM CP-odd and CP-even terms [3, 7]. The relative fractions of the CP-odd and CP-even BSM contributions to the observed Higgs boson decays are constrained, and limits on the corresponding BSM tensor couplings are derived.

This paper is organised as follows. In Sect. 2 the ATLAS detector is described. In Sect. 3 the theoretical framework used to derive the spin and parity models, as well as the parameterisation used to describe the *HVV* coupling tensor structure, are discussed. In Sect. 4, the choice of Monte Carlo generators for the simulation of signal and backgrounds is described. The analyses of fixed spin and parity hypotheses for the three decay channels and their combination are presented in Sect. 5. Individual and combined studies of the tensor structure of the *HVV* interaction are presented in Sect. 6. Concluding remarks are given in Sect. 7.

## The ATLAS detector

The ATLAS detector is described in detail in Ref. [11]. ATLAS is a multi-purpose detector with a forward-backward symmetric cylindrical geometry. It uses a right-handed coordinate system with its origin at the nominal interaction point (IP) in the centre of the detector and the *z*-axis along the beam pipe. The *x*-axis points from the IP to the centre of the LHC ring, and the *y*-axis points upward. Cylindrical coordinates $(r,\phi )$ are used in the transverse plane, $\phi$ being the azimuthal angle around the beam pipe. The pseudorapidity is defined as $\eta = -\ln \tan (\theta /2)$, where $\theta$ is the polar angle.

At small radii from the beamline, the inner detector (ID), immersed in a 2 T magnetic field produced by a thin superconducting solenoid located in front of the calorimeter, is made up of fine-granularity pixel and microstrip detectors. These silicon-based detectors cover the range $|\eta |<2.5$. A gas-filled straw-tube transition-radiation tracker (TRT) complements the silicon tracker at larger radii and also provides electron identification based on transition radiation. The electromagnetic (EM) calorimeter is a lead/liquid-argon sampling calorimeter with an accordion geometry. The EM calorimeter is divided into a barrel section covering $|\eta | <1.475$ and two end-cap sections covering $1.375<|\eta | <3.2$. For $|\eta | <2.5$ it is divided into three layers in depth, which are finely segmented in $\eta$ and $\phi$. An additional thin presampler layer, covering $|\eta | <1.8$, is used to correct for fluctuations in energy losses of particles before they reach the calorimeter. Hadronic calorimetry in the region $|\eta | <1.7$ uses steel absorbers and scintillator tiles as the active medium. Liquid argon with copper absorbers is used in the hadronic end-cap calorimeters, which cover the region $1.5<|\eta | <3.2$. A forward calorimeter using copper or tungsten absorbers with liquid argon completes the calorimeter coverage up to $|\eta | =4.9$. The muon spectrometer (MS) measures the deflection of muon trajectories with $|\eta | <2.7$, using three stations of precision drift tubes, with cathode strip chambers in the innermost layer for $|\eta | > 2.0$. The deflection is provided by a toroidal magnetic field with an integral of approximately 3 and 6 Tm in the central and end-cap regions of the ATLAS detector, respectively. The muon spectrometer is also instrumented with dedicated trigger chambers, the resistive-plate chambers in the barrel and thin-gap chambers in the end-cap, covering $|\eta | <2.4$.

## Theoretical models

In this section, the theoretical framework for the measurements of the spin and parity of the resonance is discussed. An effective field theory (EFT) approach is adopted to describe the interaction between the resonance and the SM vector bosons, following the Higgs boson characterisation model described in Refs. [3, 7]. Three possible BSM scenarios for the spin and parity of the boson are considered:

- the observed resonance is a spin-2 particle,
- the observed resonance is a pure BSM spin-0 CP-even or CP-odd Higgs boson,
- the observed resonance is a mixture of the SM spin-0 state and a BSM spin-0 CP-even or CP-odd state.

The third case would imply CP-violation in the Higgs sector. In the case of CP mixing, the Higgs boson would be a mass eigenstate, but not a CP eigenstate. In all cases, only one resonance with a mass of about 125 GeV is considered. It is also assumed that the total width of the resonance is small compared to the typical experimental resolution of the ATLAS detector (of the order of 1–2 GeV in the four-lepton and $\gamma \gamma$ final states, as documented in Ref. [12]). Interference effects between the BSM signals and SM backgrounds are neglected.

**Table 1:** Parameters of the benchmark scenarios for spin-0 boson tensor couplings used in tests (see Eq. (1)) of the fixed spin and parity models

$J^P$	Model	Values of tensor couplings			
		$\kappa _\mathrm{SM}$	$\kappa _{HVV}$	$\kappa _{AVV}$	$\alpha$
$0^+$	SM Higgs boson	1	0	0	0
$0^+_h$	BSM spin-0 CP-even	0	1	0	0
$0^-$	BSM spin-0 CP-odd	0	0	1	$\pi /2$

The EFT approach, used by the Higgs boson characterisation model, is only valid up to a certain energy scale, $\Lambda$. The models described in Ref. [7] assume that the resonance structure corresponds to one new boson ($X(J^P)$ with $J^{P} = 0^{\pm }$ or $2^+$), assuming that any other BSM particle only exists at an energy scale larger than $\Lambda$. The $\Lambda$ scale is set to 1 TeV to account for the experimental results obtained at the LHC and previous collider experiments, which do not show any evidence of new physics at lower energy scales.

The case where the observed resonance has $J^{P} = 1^{\pm }$ is not studied in this paper. The $H \rightarrow \gamma \gamma$ decay is forbidden by the Landau–Yang theorem [13, 14] for a spin-1 particle. Moreover, the spin-1 hypothesis was already studied in the previous ATLAS publication [4] in the $H \rightarrow ZZ^{*} \rightarrow 4 \ell$ and $H \rightarrow WW^{*} \rightarrow e \nu \mu \nu$ decays and excluded at a more than 99 % confidence level.

### The spin-0 hypothesis

In the spin-0 hypothesis, models with fixed spin and parity, and models with mixed SM spin-0 and BSM spin-0 CP-even and CP-odd contributions are considered. In Ref. [7], the spin-0 particle interaction with pairs of *W* or *Z* bosons is given through the following interaction Lagrangian:

1 $$\begin{aligned} \mathcal L_0^{V}= & {} \left\{ \cos (\alpha ) \kappa _\mathrm{SM}\left[ \frac{1}{2}g_{HZZ} Z_{\mu } Z^ {\mu } + g_{HWW} W^{+}_{\mu } W^ {- \mu }\right] \right. \nonumber \\&\left. -\frac{1}{4}\frac{1}{\Lambda }\left[ \cos (\alpha ) \kappa _{HZZ} Z_{\mu \nu }Z^{\mu \nu }+ \sin (\alpha ) \kappa _{AZZ} Z_{\mu \nu } \tilde{Z}^{\mu \nu }\right] \right. \nonumber \\&\left. -\frac{1}{2} \frac{1}{\Lambda } \left[ \cos (\alpha ) \kappa _{HWW} W^+_{\mu \nu }W^{-\mu \nu }\right. \right. \nonumber \\&\left. \left. + \sin (\alpha ) \kappa _{AWW} W^+_{\mu \nu } \tilde{W}^{ - \mu \nu }\right] \right\} X_0. \end{aligned}$$

Here $V^{\mu }$ represents the vector-boson field ${(V=Z,W^{\pm }})$, the $V^{\mu \nu }$ are the reduced field tensors and the dual tensor is defined as $\tilde{V}^{\mu \nu }= \frac{1}{2}\varepsilon ^{\mu \nu \rho \sigma }V_{\rho \sigma }$. The symbol $\Lambda$ denotes the EFT energy scale. The symbols $\kappa _\mathrm{SM}$, $\kappa _{HVV}$ and $\kappa _{AVV}$ denote the coupling constants corresponding to the interaction of the SM, BSM CP-even or BSM CP-odd spin-0 particle, represented by the $X_0$ field, with *ZZ* or *WW* pairs. To ensure that the Lagrangian terms are Hermitian, these couplings are assumed to be real. The mixing angle $\alpha$ allows for production of CP-mixed states and implies CP-violation for $\alpha \ne 0$ and $\alpha \ne \pi$, provided the corresponding coupling constants are non-vanishing. The SM couplings, $g_{HVV}$, are proportional to the square of the vector boson masses: $g_{HVV} \propto m^{2}_{V}$. Other higher-order operators described in Ref. [7], namely the derivative operators, are not included in Eq. (1) and have been neglected in this analysis since they induce modifications of the discriminant variables well below the sensitivity achievable with the available data sample.

As already mentioned, for the spin-0 studies the SM Higgs boson hypothesis is compared to two alternatives: the CP-odd $J^P=0^-$ and the BSM CP-even $J^P=0^+_h$ hypotheses. All three models are obtained by selecting the corresponding parts of the Lagrangian described in Eq. (1) while setting all other contributions to zero. The values of the couplings corresponding to the different spin-0 models are listed in Table 1.

The investigation of the tensor structure of the *HVV* interaction is based on the assumption that the observed particle has spin zero. Following the parameterisation defined in Eq. (1), scenarios are considered where only one CP-odd or one CP-even BSM contribution at a time is present in addition to the SM contribution. To quantify the presence of BSM contributions in $H \rightarrow ZZ^{*}$ and $H \rightarrow WW^{*}$ decays, the ratios of couplings $(\tilde{\kappa }_{AVV}/\kappa _\mathrm{SM})\cdot \tan {\alpha }$ and $\tilde{\kappa }_{HVV}/\kappa _\mathrm{SM}$ are measured. Here $\tilde{\kappa }_{AVV}$ and $\tilde{\kappa }_{HVV}$ are defined as follows:

2 $$\begin{aligned} \tilde{\kappa }_{ AVV}=\frac{1}{4} \frac{ \mathrm{v} }{\Lambda } \kappa _{AVV}\quad \mathrm{and}\quad \tilde{\kappa }_{HVV}=\frac{1}{4} \frac{ \mathrm{v} }{\Lambda } \kappa _{HVV}, \end{aligned}$$

where $\mathrm{v}$ is the vacuum expectation value [15] of the SM Higgs field.

The mixing parameters $(\tilde{\kappa }_{AVV}/\kappa _\mathrm{SM})\cdot \tan {\alpha }$ and $\tilde{\kappa }_{HVV}/\kappa _\mathrm{SM}$ correspond to the ratios of tensor couplings $g_4/g_1$ and $g_2/g_1$ proposed in the anomalous coupling approach described in Refs. [9, 10]. To compare the results obtained in this analysis to other existing studies, the final results are also expressed in terms of the effective cross-section fractions $(f_{g2},\phi _{g2})$ and $(f_{g4},\phi _{g4})$ proposed in Refs. [3, 9, 10]. Further details of these conversions are given in Appendix A.

The BSM terms described in Eq. (1) are also expected to change the relative contributions of the vector-boson fusion (VBF) and vector-boson associated production (*VH*) processes with respect to the gluon-fusion (ggF) production process, which is predicted to be the main production mode for the SM Higgs boson at the LHC. For large values of the BSM couplings, at the LHC energies, the VBF production mode can have a cross section that is comparable to the ggF process [16]. This study uses only kinematic properties of particles from $H \rightarrow V V^{*}$ decays to derive information on the CP nature of the Higgs boson. The use of the signal rate information for different production modes, in the context of the EFT analysis, may increase the sensitivity to the BSM couplings at the cost of a loss in generality. For example the ratio of the VBF and *VH* production modes with respect to the ggF one can be changed by a large amount for non-vanishing values of the BSM couplings. In the studies presented in this paper the predictions of the signal rates are not used to constrain the BSM couplings.

As described in Sect. 6.2, only events with no reconstructed jets (the 0-jet category) are used in the $H \rightarrow WW^{*} \rightarrow e \nu \mu \nu$ analysis for the studies of the tensor structure; hence this analysis has little sensitivity to the VBF production mode. The $H \rightarrow ZZ^{*} \rightarrow 4 \ell$ analysis also has little sensitivity to this production mode since it is mainly based on variables related to the four-lepton kinematics. The Boosted Decision Tree (BDT) algorithm [17] used to discriminate signals from the $ZZ^{*}$ background, described in Sects. 5.4 and 6.3, includes the transverse momentum of the four-lepton system and is trained on simulated samples of ggF-produced signals. An enhancement of the VBF production mode would improve the separation between background and signal since it predicts larger values of the transverse momentum spectrum for events produced via VBF than via ggF [3].

### The spin-2 hypothesis

In the Higgs boson characterisation model [7], the description of the interaction of a spin-2 particle with fermions and vector bosons is described by the following Lagrangian:

3 $$\begin{aligned} \mathcal L_2 = - \frac{1}{\Lambda }\left[ \sum _V \kappa _V \mathcal{T}^V_{\mu \nu } X^{\mu \nu } + \sum _f \kappa _f \mathcal{T}^f_{\mu \nu } X^{\mu \nu } \right] . \end{aligned}$$

The spin-2 tensor field $X^{\mu \nu }$ is chosen to interact with the energy-momentum tensors, $\mathcal{T}^V_{\mu \nu }$ and $\mathcal{T}^f_{\mu \nu }$, of any vector boson *V* and fermion *f*, as inspired by gravitation theories. The strength of each interaction is determined by the couplings $\kappa _V$ and $\kappa _f$. In the simplest formulation, all couplings are equal. This scenario is referred to as universal couplings (UC), while scenarios with different values of the couplings are referred to as non-universal couplings (non-UC). In the UC scenario, the production of a spin-2 particle in *pp* collisions is expected to be dominated by QCD processes, with negligible contributions from electroweak (EW) processes (i.e. from processes involving EW boson propagators). Simulation studies based on MadGraph5_aMC@NLO [16], which implements the Lagrangian described in Eq. (3), predict for the production cross section in the UC scenario $\sigma _{\mathrm {EW}}/\sigma _{\mathrm {QCD}}\simeq 3 \times 10^{-4}$. These studies also show that EW production of the spin-2 resonance would occur mainly in association with a massive EW boson (*WX*, *ZX*). Present observations do not show a dominant *VH* production mechanism, hence suggesting that $\sigma _{\mathrm {EW}}$ is significantly smaller than $\sigma _{\mathrm {QCD}}$. This paper considers only QCD production for all the spin-2 benchmark scenarios.

The UC models predict a branching ratio of about 5 % to photon pairs and negligible branching ratios to massive EW gauge boson pairs, $WW^*$ and $ZZ^*$. This prediction is disfavoured by the experimental measurements [18–20] and therefore the equality between all couplings $\kappa$ cannot hold. In the benchmark scenarios studied in this paper, each of the couplings $\kappa _W$, $\kappa _Z$, and $\kappa _\gamma$ is assumed to be independent of all the other couplings. In the following, the UC scenario only refers to $\kappa _{q} = \kappa _{g}$, without implying the equality for the other $\kappa$ values.

The simplest QCD production processes, $gg\rightarrow X$ and $q\bar{q} \rightarrow X$ (where *q* refers to light quarks), yield different polarisations for the spin-2 particle *X*, and hence different angular distributions of its decay products. These mechanisms are considered in the model of a graviton-like tensor with minimal couplings proposed in Refs. [9, 10], which has been studied experimentally in Ref. [4]. The EFT Lagrangian, however, also allows for more complex processes with emission of one or more additional partons. For instance, processes with one-parton emission, like $qg\rightarrow qX$ and $~\bar{q}g\rightarrow \bar{q}X$, can produce a spin-2 state through either a *qqX* or a *ggX* vertex. When two partons are emitted, as in $gg\rightarrow q\bar{q}X$ or $q\bar{q}\rightarrow q\bar{q}X$, the spin-2 production may occur through *qqX* or *ggX* vertices, respectively, such that the polarisation of *X* is not uniquely determined by the initial state. Moreover, the EFT also allows for four-leg vertices like *qqgX*. These additional diagrams effectively change the polarisation of the particle *X*, compared to what is assumed by the model in Refs. [9, 10]. As a consequence, the angular distributions of the decay products become harder to separate from those expected for a scalar resonance.

The QCD production of a spin-2 particle is driven by the values of the couplings $\kappa _g,~\kappa _q$. Presently, there are no experimental constraints on the ratio $\kappa _q/\kappa _g$ from observed decay modes, since the separation of jets initiated by gluons or by light quarks is experimentally difficult and has not yet been attempted in Higgs boson studies. The ratio $\kappa _q/\kappa _g$ can thus be regarded as a free parameter. When $\kappa _q\ne \kappa _g$, the spin-2 model predicts an enhancement of the tail of the distribution of the transverse momentum, $p_{\text {T}} ^X$, of the spin-2 particle. Such a high-$p_{\text {T}} ^X$ tail is not present for the $\kappa _q = \kappa _g$ (UC) case. As stated before, however, the EFTs are valid only up to some energy scale, $\Lambda$. At higher energies, new physics phenomena are expected to enter to regularise the anomalous ultra-violet behaviour.

In the present analysis, a selection $p_{\text {T}} ^X<300$ GeV is applied when investigating non-UC scenarios, $\kappa _q\ne \kappa _g$. In addition, for the non-UC scenarios, analyses using a tighter selection $p_{\text {T}} ^X<125~\text {GeV}$ are also performed. This is a conservative choice for the $p_{\text {T}} ^{X}$ selection, as the EFT must describe the physics at least up to the mass of the observed resonance. It has been verified that the choice of the $p_{\text {T}} ^{X}$ selection does not affect the results for the UC scenario. Even assuming the $p_{\text {T}} ^X<300$ GeV selection, some choices of $\kappa _q/\kappa _g$ produce high-$p_{\text {T}} ^X$ tails incompatible with the observed differential distribution reported in Refs. [21, 22]. For this reason the investigated range of the $\kappa _q/\kappa _g$ ratio is limited to between zero and two. The spin-2 scenarios considered in this study are presented in Table 2. The $\kappa _{q} = \kappa _{g}$ model is referred to hereafter as the UC scenario. The $\kappa _q=0$ case implies a negligible coupling to light quarks, whereas the $\kappa _q=2\kappa _g$ case is an alternative scenario with an enhanced coupling to quarks.

**Table 2:** Choices of the couplings to quarks $\kappa _{q}$ and to gluons $\kappa _{g}$ studied for the spin-2 benchmark scenarios. The values of the selection criteria applied to the transverse momentum $p_{\text {T}} ^X$ of the spin-2 resonance are also shown. For the UC scenario no $p_{\text {T}} ^X$ selection is applied

Values of spin-2 quark and gluon couplings		$p_{\text {T}} ^X$ selections ($\text {GeV}$)	
$\kappa _q=\kappa _g$	Universal couplings	–	–
$\kappa _q=0$	Low light-quark fraction	${<}300$	${<}125$
$\kappa _q=2\kappa _g$	Low gluon fraction	${<}300$	${<}125$

## Data and simulated samples

The data presented in this paper were recorded by the ATLAS detector during the 2012 LHC run with proton–proton collisions at a centre-of-mass energy of 8 TeV, and correspond to an integrated luminosity of 20.3 fb$^{-1}$. For the $H \rightarrow \gamma \gamma$ and $H \rightarrow ZZ^{*} \rightarrow 4 \ell$ channels, the data collected in 2011 at a centre-of-mass energy of 7 TeV corresponding to an integrated luminosity of 4.5 fb$^{-1}$, are also used. Data quality requirements are applied to reject events recorded when the relevant detector components were not operating correctly. More than 90 % of the recorded luminosity is used in these studies. The trigger requirements used to collect the data analysed in this paper are the same as those described in previous publications [18–20]. They are only briefly recalled in the following sections.

The Monte Carlo (MC) samples for the backgrounds and for the SM Higgs boson signal are the same as those used for the analyses described in Refs. [18–20], whereas new non-SM signal samples have been simulated. An overview of the signal samples is given in Sect. 4.1.

The effects of the underlying event and of additional minimum-bias interactions occurring in the same or neighbouring bunch crossings, referred to as pile-up in the following, are modelled with Pythia 8 [23]. The ATLAS detector response is simulated [24] using either Geant 4 [25] alone or combined with a parameterised Geant 4-based calorimeter simulation [26].

### SM Higgs boson and BSM signal samples

The SM Higgs boson ggF production for all analyses is modelled using the Powheg-Box [27] generator at next-to-leading order (NLO), interfaced to Pythia 8 for parton showering and hadronisation and to simulate multi-parton interactions. To improve the modelling of the SM Higgs boson $p_{\text {T}}$, a reweighting procedure is applied. This procedure applies a weight depending on the $p_{\text {T}}$ of the Higgs boson to each event. The weights are chosen in order to reproduce the prediction of the next-to-next-to-leading-order (NNLO) and next-to-next-to-leading-logarithms (NNLL) dynamic-scale calculation given by the hres2.1 program [28, 29].

For the $H \rightarrow \gamma \gamma$ analysis, the signal samples are generated at several values of the Higgs boson mass $m_H$ around 125 GeV. The samples are used to obtain a parameterisation of the signal yields and of the invariant mass distribution of the two-photon system as continuous functions of $m_H$ (both inclusively and for each category in the analysis, as described in Sect. 5.2). The spin-2 samples are generated using the MadGraph5_aMC@NLO [16] program with LO accuracy for zero, one, and two additional partons, and with subsequent matching of the matrix-element calculation with a model of the parton shower, underlying event and hadronisation, using Pythia 6 [30].

In the $H \rightarrow ZZ^{*} \rightarrow 4 \ell$ analysis the signal samples representing the production and decay of Higgs bosons with spin-0 and different parities are generated as follows. The SM Higgs boson production via gluon fusion at the mass $m_H=125.5$ GeV is simulated using the Powheg-Box generator. For the non-SM signals, the decays of the generated Higgs bosons are simulated, according to the Higgs boson parity assumptions, using the JHU [9, 10] MC generator at leading order (LO). The spin-2 samples are generated using the MadGraph5_aMC@NLO MC generator, as for the $H \rightarrow \gamma \gamma$ analysis.

For the $H \rightarrow WW^{*} \rightarrow e \nu \mu \nu$ analysis, the SM Higgs boson signal is generated at $m_H = 125$ GeV using the Powheg-Box Monte Carlo generator. The spin-0 BSM signal samples are generated using MadGraph5_aMC@NLO. The signal samples representing the production and decay of Higgs bosons with spin-2 are generated using the MadGraph5_aMC@NLO MC generator, as for the $H \rightarrow \gamma \gamma$ analysis.

For studies of the tensor structure of the *HVV* decay, all simulated signal samples are obtained by using the matrix element (ME) reweighting method applied, as explained in the following, to a sample generated with non-zero values of the BSM couplings. The reweighting procedure is validated against samples produced at different values of the couplings, to ensure that the distributions of the CP-sensitive final-state observables and of their correlations are reproduced correctly. For the $H \rightarrow ZZ^{*} \rightarrow 4 \ell$ analysis, the MC production is only performed for one set of tensor couplings: $g_1=1$, $g_2=1+i$, $g_4= 1+i$. All other configurations of couplings are obtained by reweighting this sample at generator level. The ratios of the corresponding squares of ME values calculated at LO are used as weights. To calculate these ME values, the JHUGenME [10] program is used. In the $H \rightarrow WW^{*} \rightarrow e \nu \mu \nu$ analysis, only one MC sample is generated, using MadGraph5_aMC@NLO with parameters $\kappa _\mathrm{SM} =1$, $\kappa _{AWW} = 2$, $\kappa _{HWW} = 2$, $\cos (\alpha ) = 0.3$, and all other samples are obtained from it by reweighting the events on the basis of the ME amplitudes.

In all the analyses presented in this paper, the mass of the Higgs boson is fixed to 125.4 GeV  [12].

### Background samples

The MC simulated samples for the backgrounds, as well as for the determinations of the corresponding cross sections, are the same as those adopted in Refs. [18–20]. In the $H \rightarrow \gamma \gamma$ analysis, the background is dominated by prompt $\gamma \gamma$ events, with smaller contributions from $\gamma -$jet events. For the $H \rightarrow ZZ^{*} \rightarrow 4 \ell$ analysis, the major background is the non-resonant $ZZ^{*}$ process, with minor contributions from the $t\bar{t}$ and *Z*+jets processes. For the $H \rightarrow WW^{*} \rightarrow e \nu \mu \nu$ analysis, the dominant backgrounds are non-resonant *W* boson pair (*WW*) production, $t\bar{t}$ and single-top-quark production, and the $Z/\gamma ^{*}$ process followed by the decay to $\tau \tau$ final states.

## Tests of fixed spin and parity hypotheses

The $H \rightarrow \gamma \gamma$ and $H \rightarrow ZZ^{*} \rightarrow 4 \ell$ analyses are improved with respect to the previous ATLAS publication of Ref. [4]. These analyses are described in some detail in the following subsections. The spin and parity analysis in the $H \rightarrow WW^{*} \rightarrow e \nu \mu \nu$ channel has also been improved, as discussed in detail in a separate publication [8]. In the following, only a brief overview of this analysis is given. The expected and observed results of the individual channels and of their combination are presented in Sect. 5.5.

### Statistical treatment

The analyses rely on discriminant observables chosen to be sensitive to the spin and parity of the signal.

**Fig. 1:**
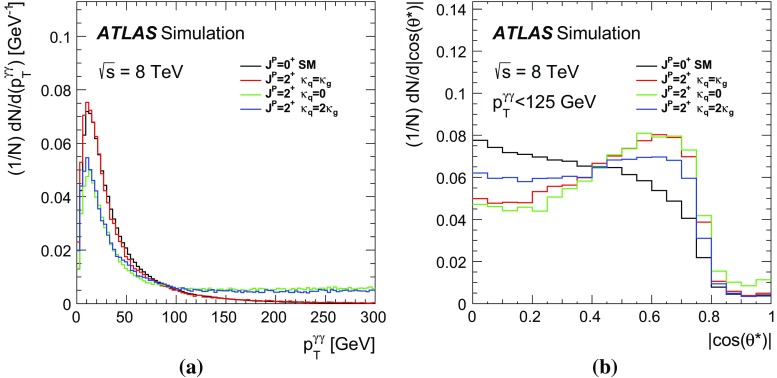
Expected distributions of kinematic variables sensitive to the spin of the resonance considered in the $H \rightarrow \gamma \gamma$ analysis, **a** transverse momentum of the $\gamma \gamma$ system $p_{\text {T}} ^{\gamma \gamma }$ and **b** the production angle of the two photons in the Collins–Soper frame $|\cos \theta ^*|$, for a SM Higgs boson and for spin-2 particles with three different choices of the QCD couplings

A likelihood function, $\mathcal{L}(\mathrm{data}~|~J^P, \mu , \vec {\theta })$, that depends on the spin-parity assumption of the signal is constructed as a product of conditional probabilities over binned distributions of the discriminant observables in each channel:

4 $$\begin{aligned} \mathcal {L}(\mathrm{data}~|~ J^P, \mu , \vec {\theta })= & {} \displaystyle \prod _{j}^{N_{\mathrm {chann.}}} \prod _{i}^{N_{\mathrm {bins}}} P\big (N_{i,j} ~| ~\mu _{j} \cdot S^{(J^P)}_{i, j}(\vec {\theta }) \nonumber \\&+ B_{i,j}(\vec {\theta }) \big ) \cdot \mathcal {A}_{j}(\vec {\theta }) \;, \end{aligned}$$

where $\mu _{j}$ represents the parameter associated with the signal rate normalised to the SM prediction in each channel *j*.^3^ The symbol $\vec {\theta }$ represents all nuisance parameters. The likelihood function is a product of Poisson distributions *P* corresponding to the observation of $N_{i,j}$ events in each bin *i* of the discriminant observables, given the expectations for the signal, $S_{i,j}^{(J^P)}(\vec {\theta })$, and for the background, $B_{i,j}(\vec {\theta })$. Some of the nuisance parameters are constrained by auxiliary measurements. Corresponding constraints are represented by the functions $\mathcal {A}_{j}(\vec {\theta })$.

While the couplings are predicted for the SM Higgs boson, they are not known *a priori* for the alternative hypotheses, defined as $J^{P}_{\mathrm {alt} }$, as discussed in Sect. 3. In order to be insensitive to assumptions on the couplings of the non-SM resonance (the alternative hypotheses) to SM particles, the numbers of signal events in each channel, for each different LHC centre-of-mass energy and for each tested hypothesis, are treated as independent parameters in the likelihood and fitted to the data when deriving results on the spin and parity hypotheses.

The test statistic $\tilde{q}$ used to distinguish between the two spin-parity hypotheses is based on a ratio of profiled likelihoods [31, 32]:

5 $$\begin{aligned} \tilde{q} = \log \frac{\mathcal {L}\left( J^{P}_{\mathrm {SM} }, \hat{\hat{\mu }}_{J^{P}_{\mathrm {SM} }}, \hat{\hat{\theta }}_{J^{P}_{\mathrm {SM} }}\right) }{\mathcal {L}\left( J^{P}_{\mathrm {alt} }, \hat{\hat{\mu }}_{J^{P}_{\mathrm {alt} }}, \hat{\hat{\theta }}_{J^{P}_{\mathrm {alt} }} \right) }, \end{aligned}$$

where $$\mathcal {L}(J^P, \hat{\hat{\mu }}_{J^P}, \hat{\hat{\theta }}_{J^P})$$ is the maximum-likelihood estimator, evaluated under either the SM $$J^{P}_{\mathrm {SM} }= 0^{+}$$ or the alternative $$J^{P}_{\mathrm {alt} }$$ spin-parity hypothesis. The parameters $$\hat{\hat{\mu }}_{J^P}$$ and $$\hat{\hat{\theta }}_{J^P}$$ represent the values of the signal strength and nuisance parameters fitted to the data under each spin and parity hypothesis. The distributions of the test statistic for both hypotheses are obtained using ensemble tests of MC pseudo-experiments. For each hypothesis test, about 70, 000 pseudo-experiments were generated. The generation of the pseudo-experiments uses the numbers of signal and background events in each channel obtained from maximum-likelihood fits to data. In the fits of each pseudo-experiment, these and all other nuisance parameters are profiled, i.e. fitted to the value that maximises the likelihood for each value of the parameter of interest. When generating the distributions of the test statistic for a given spin-parity hypothesis, the expectation values of the signal strengths are fixed to those obtained in the fit to the data under the same spin-parity assumption. The distributions of $$\tilde{q}$$ are used to determine the corresponding *p*-values $$p(J^{P}_{\mathrm {SM} })=p^\mathrm{SM}$$ and $$p(J^{P}_{\mathrm {alt} })=p^\mathrm{alt}$$. For a tested hypothesis $$J^{P}_{\mathrm {alt} }$$, the observed (expected) *p*-values are obtained by integrating the corresponding distributions of the test statistic above the observed value of $$\tilde{q}$$ (above the median of the $$J^{P}_{\mathrm {SM} }$$$$\tilde{q}$$ distribution). When the measured data are in agreement with the tested hypothesis, the observed value of $$\tilde{q}$$ is distributed such that all *p*-values are equally probable.

Very small values of the integral of the distribution of the test statistic for the $J^{P}_{\mathrm {alt} }$ hypothesis, corresponding to large values of $\tilde{q}$, are interpreted as the data being in disagreement with the tested hypothesis in favour of the SM hypothesis.

**Fig. 2:**
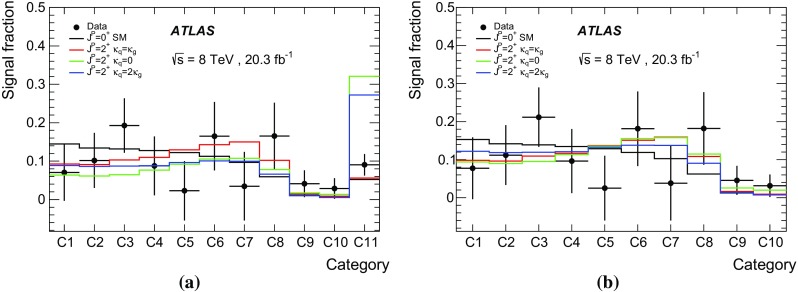
Observed signal fraction per category for the $H \rightarrow \gamma \gamma$ analysis, and comparison to expected values for a SM Higgs boson and for a spin-2 particle with different choices of QCD couplings. **a** The 11 categories described in the text are displayed, corresponding to the $p_{\text {T}} ^{\gamma \gamma }<300~\text {GeV}$ selection; **b** the high-$p_{\text {T}} ^{\gamma \gamma }$ category is discarded and the signal fractions are renormalised over the 10 remaining categories, corresponding to the $p_{\text {T}} ^{\gamma \gamma }<125~\text {GeV}$ selection

The exclusion of the alternative $J^{P}_{\mathrm {alt} }$ hypothesis in favour of the SM $J^{P}_{\mathrm {SM} }$ hypothesis is evaluated in terms of the modified confidence level $\mathrm{CL}_\mathrm{s}(J^{P}_{\mathrm {alt} })$, defined as [33]:

6 $$\begin{aligned} \mathrm{CL}_\mathrm{s}(J^{P}_{\mathrm {alt} }) = \frac{p (J^{P}_{\mathrm {alt} })}{ 1- p (J^{P}_{\mathrm {SM} })}\;. \end{aligned}$$

### Spin analysis in the $H \rightarrow \gamma \gamma$ channel 

The analysis in the $H \rightarrow \gamma \gamma$ channel is sensitive to a possible spin-2 state. Since the spin-2 models investigated in the present paper are different from those assumed in Ref. [4], the analysis has been redesigned, to improve its sensitivity to the new models.

The selection of $H\rightarrow \gamma \gamma$ candidate events is based on the procedure of other recent ATLAS $H \rightarrow \gamma \gamma$ analyses (see for example Ref. [20]). Events are selected if they satisfy a diphoton trigger criterion requiring loose photon identification, with transverse momentum $p_{\text {T}}$ thresholds of 35 and 25 GeV for the photon with the highest ($\gamma _1$) and second-highest ($\gamma _2$) $p_{\text {T}}$, respectively. During the offline selection two photons are further required to be in a fiducial pseudorapidity region, defined by $|\eta ^\gamma |<2.37$, where the barrel/end-cap transition region $1.37 < |\eta ^\gamma | < 1.56$ is excluded. The transverse momentum of the photons must satisfy $p_{\text {T}} ^{\gamma _1}>0.35\cdot m_{\gamma \gamma }$ and $p_{\text {T}} ^{\gamma _2}>0.25\cdot m_{\gamma \gamma }$, and only events with a diphoton invariant mass $m_{\gamma \gamma }$ between 105 and 160 GeV are retained. For the events passing this selection, a further requirement is applied on the diphoton transverse momentum, $p_{\text {T}} ^{\gamma \gamma }<300~\text {GeV}$, motivated by the assumed validity limit of the spin-2 EFT model, as explained in Sect. 3. After this selection, 17,220 events are left at a centre-of-mass energy $\sqrt{s}=7~\text {TeV}$ and 94,540 events at $\sqrt{s}=8~\text {TeV}$.

Kinematic variables sensitive to the spin of the resonance are the diphoton transverse momentum $p_{\text {T}} ^{\gamma \gamma }$ and the production angle of the two photons, measured in the Collins–Soper frame [34]:

7 $$\begin{aligned} |\cos \theta ^*|\ = \frac{|\sinh (\Delta \eta ^{\gamma \gamma }) |}{\sqrt{1+ ( p_{\text {T}} ^{\gamma \gamma }/m_{\gamma \gamma })^2}}\frac{2p_{\text {T}} ^{\gamma _1}p_{\text {T}} ^{\gamma _2}}{m_{\gamma \gamma }^2}, \end{aligned}$$

where $\Delta \eta ^{\gamma \gamma }$ is the separation in pseudorapidity of the two photons.

The predicted distributions of these variables, for events passing the selection, are shown in Fig. 1, for a SM Higgs boson and for a spin-2 particle with different QCD couplings. For the $\kappa _q\ne \kappa _g$ cases, the enhanced high-$p_{\text {T}} ^{\gamma \gamma }$ tail offers the best discrimination, whereas for $\kappa _q=\kappa _g$ the most sensitive variable is $|\cos \theta ^*|$.

To exploit the signal distribution in both $p_{\text {T}} ^{\gamma \gamma }$ and $|\cos \theta ^*|$, the selected events are divided into 11 mutually exclusive categories: 10 categories (labelled from C1 to C10) collect events with $p_{\text {T}} ^{\gamma \gamma }<125~\text {GeV}$, divided into 10 bins of equal size in $|\cos \theta ^*|$, while the 11th category (labelled C11) groups all events with $p_{\text {T}} ^{\gamma \gamma }\ge 125~\text {GeV}$. As described in Sect. 3, for the non-UC spin-2 models the analysis is performed with two $p_{\text {T}} ^{\gamma \gamma }$ selections, namely $p_{\text {T}} ^{\gamma \gamma }<300~\text {GeV}$ and $p_{\text {T}} ^{\gamma \gamma }<125~\text {GeV}$: the latter case corresponds to not using the 11th category.

The number of signal events above the continuum background can be estimated through a fit to the observed $m_{\gamma \gamma }$ distribution in each category. The $m_{\gamma \gamma }$ distribution is modelled in each category as the sum of one-dimensional probability density functions (pdf) for signal and background distributions:

8 $$\begin{aligned} f^{[c]}(m_{\gamma \gamma }|J)= & {} \frac{n_B^{[c]}f_B^{[c]}(m_{\gamma \gamma })+(n_J^{[c]}+n_\mathrm{bias}^{[c]})f_S^{[c]}(m_{\gamma \gamma })}{n_B^{[c]}+n_J^{[c]}+n_\mathrm{bias}^{[c]}},\nonumber \\ \end{aligned}$$

where *J* is the spin hypothesis, $n_B^{[c]}$ and $n_J^{[c]}$ are the background and the signal yield in category *c*, and $f_B^{[c]}(m_{\gamma \gamma }),~f_S^{[c]}(m_{\gamma \gamma })$ are the $m_{\gamma \gamma }$ pdfs for the background and the signal, respectively. The signal pdf $f_S^{[c]}(m_{\gamma \gamma })$ is modelled as a weighted sum of a Crystal Ball function, describing the core and the lower mass tail, and of a Gaussian component that improves the description of the tail for higher mass values. For each category, $f_S^{[c]}(m_{\gamma \gamma })$ is fitted to the simulated $m_{\gamma \gamma }$ distribution of the SM Higgs boson and verified to be consistent also with the spin-2 models. The background pdf $f_B^{[c]}(m_{\gamma \gamma })$ is empirically modelled as an exponential of a first- or second-degree polynomial. The choice of such a parameterisation can induce a bias (“spurious signal”) in the fitted signal yield, which is accounted for by the term $n_\mathrm{bias}^{[c]}$. The size of the expected bias is determined as described in Refs. [20, 22], and ranges between 0.6 and 4 events, depending on the category (with the signal ranging from 15 to more than 100 events). In the statistical analysis, $n_\mathrm{bias}^{[c]}$ is constrained for each category by multiplying the likelihood function by a Gaussian function centred at zero and with a width determined by the size of the expected bias.

Defining $n_S$ as the total signal yield (summed over all categories), the expected fraction of signal events belonging to each category, $\Phi _J^{[c]}\equiv \frac{n_J^{[c]}}{n_S}$, depends on the spin hypothesis *J*. The values of $\Phi _J^{[c]}$ extracted from the data can be compared to their expected values for each spin hypothesis, as shown in Fig. 2 for the data collected at $\sqrt{s}=8~\text {TeV}$.

For the non-UC scenario the 11th (high-$p_{\text {T}} ^{\gamma \gamma }$) category provides strong discrimination power against the non-SM hypothesis, as visible in Fig. 2a.

To discriminate between the SM spin-0 ($J^{P}_{\mathrm {SM} }= 0^{+}$) and alternative spin-2 hypotheses ($J^{P}_{\mathrm {alt} }$), two likelihood functions $\mathcal{L}_{J^{P}_{\mathrm {SM} }},~\mathcal{L}_{J^{P}_{\mathrm {alt} }}$ are built, following the general approach described in Eq. (4):

9 $$\begin{aligned} -\ln \mathcal{L}_J= & {} \sum _{c} \left\{ \left( n_B^{[c]}+n_S\Phi _J^{[c]}+n_\mathrm{bias}^{[c]}\right) \right. \nonumber \\&- \sum _{e\in [c]} \ln ~\Big [ n_B^{[c]}f_B^{[c]}\left( m_{\gamma \gamma }^{(e)}\right) \nonumber \\&\left. +\left( n_S\Phi _J^{[c]}+n_\mathrm{bias}^{[c]}\right) f_S^{[c]}(m_{\gamma \gamma }^{(e)})\Big ] \right\} \end{aligned}$$

where $\sum _{c}$ runs over all categories and $\sum _{e\in [c]}$ runs over all events in category *c*. The total signal yield $n_S$ is a free parameter in the likelihood model. The spin hypothesis being tested enters the likelihood function through the fractions of signal per category, $\Phi _J^{[c]}$.

Several systematic uncertainties enter this model. They are implemented for each spin hypothesis as nuisance parameters, $\theta _J$, constrained by multiplicative Gaussian terms in the likelihood function (not included in Eq. (9) for simplicity).

The signal fractions, $\Phi _J^{[c]}$, for the SM Higgs boson are affected by uncertainties on the $p_{\text {T}}$ spectrum of the resonance and on the size of the interference between the resonance and continuum production. The former is computed as described in Ref. [20]. The relative impact on the signal fractions is less than $\pm 1\,\%$ for categories 1 to 8 ($p_{\text {T}} ^{\gamma \gamma }<125~\text {GeV}$ and $|\cos \theta ^*|<0.8$), and becomes as large as $\pm 13\,\%$ for categories 10 and 11. The correction for the interference is evaluated according to Refs. [35, 36]. The systematic uncertainty is conservatively assumed to equal the correction itself, and its relative impact ranges between $\pm 0.1\,\%$ and $\pm 1.8\,\%$.

No systematic uncertainty is assigned to the simulated $p_{\text {T}} ^{X}$ distribution of the spin-2 models. The effect of the interference between the resonance and continuum production is essentially not known, as it depends on the width, $\Gamma _X$, of the resonance, which is unknown. The results presented here only hold under the assumption of a narrow width for the resonance, such that interference effects can be neglected.

Additional systematic uncertainties come from the calibration of the photon energy scale and energy resolution and affect the signal parameterisation $f_S^{[c]}$. These uncertainties are evaluated as described in Ref. [12].

### Spin and parity analysis in the $H \rightarrow WW^{*} \rightarrow e \nu \mu \nu$ channel

The analysis of the spin and parity in the $H \rightarrow WW^{*} \rightarrow e \nu \mu \nu$ channel is described in detail in a separate publication [8]. In the following a brief summary is provided. The selection is restricted to events containing two charged leptons of different flavour (one electron and one muon). The $e \nu \mu \nu$ channel is the most sensitive one [19]. The same-flavour channels ($e \nu e \nu$ and $\mu \nu \mu \nu$) are not expected to add much in terms of sensitivity due to the presence of large backgrounds that cannot be removed without greatly reducing the acceptance of the alternative models considered in this analysis. The leading lepton is required to have $p_{\text {T}} >22$ GeV and to match the object reconstructed by the trigger, while the sub-leading lepton needs to have $p_{\text {T}} >15$ GeV. While the spin-0 analyses select only events with no jets in the final state (no observed jets with $p_{\text {T}} >25$ GeV within $|\eta | < 2.5$ or with $p_{\text {T}} >30$ GeV within ${2.5 < |\eta | < 4.5}$), the spin-2 analysis enlarges the acceptance by allowing for zero or one jet (selected according to the above mentioned criteria).

The major sources of background after the dilepton selection are $$Z/\gamma ^{*}$$+jets (Drell–Yan) events, diboson ($$WW, WZ/ \gamma ^*, ZZ/ \gamma ^{*}$$), top-quark ($$t\bar{t}$$ and single top) production, and *W* bosons produced in association with hadronic jets (*W*+jets), where a jet is misidentified as a lepton. The contribution from misidentified leptons is significantly reduced by the requirement of two high-$$p_{\text {T}} $$ isolated leptons. Drell–Yan events are suppressed through requirements on some of the dilepton variables^4^ ($$p_{\text {T}} ^{\ell \ell }$$$$> 20$$ GeV, $$\Delta \phi _{\ell \ell }$$$$< 2.8$$), while a cut on $$m_{\ell \ell }$$ ($$m_{\ell \ell }< 80$$ GeV) targets the *WW* background. For alternative spin models with non-universal couplings, as discussed in Sect. 3, an additional upper bound is imposed on the Higgs boson $$p_{\text {T}} $$, reconstructed as the transverse component of the vector sum of the momenta of the two charged leptons and the missing transverse momentum. Additionally, for events containing one jet, which include substantial top-quark and *W*+jets backgrounds, *b*-jet and $$Z\rightarrow \tau ^+\tau ^-$$ vetoes are applied, together with transverse mass requirements: the larger of the transverse masses of the two *W* bosons (each computed using the corresponding lepton and the missing transverse momentum) in the event is required to be larger than 50 GeV, while the total transverse mass of the *WW* system (defined with the two leptons and the missing transverse momentum) is required to be below 150 GeV.

Control regions (CRs) are defined for the *WW*, top-quark and Drell–Yan backgrounds, which are the most important ones after the topological selection described above. The CRs are used to normalise the background event yields with a fit to the rates observed in data. The simulation is then used to transfer these normalisations to the signal region (SR). The $W$+jets background is estimated entirely from data, while non-*WW* diboson backgrounds are estimated using MC simulation and cross-checked in a validation region.

After the signal region selection, 4730 and 1569 candidate events are found in data in the 0-jet and 1-jet categories, respectively. For the latter category, the number decreases to 1567 and 1511 events when applying a selection on the Higgs boson $p_{\text {T}}$ of less than 300 GeV and less than 125 GeV, respectively. In total 218 (77) events are expected from a SM Higgs boson signal in the 0-jet (1-jet) category, while about 4390 (1413) events are expected for the total background.

A BDT algorithm is used in both the fixed spin hypothesis tests and the tensor structure analyses. For spin-2 studies, the strategy follows the one adopted in Ref. [4], with the main difference being that the 1-jet channel has been added. Two BDT discriminants are trained to distinguish between the SM hypothesis and the background (BDT$_0$), and the alternative spin hypothesis and the background (BDT$_2$). Both BDTs employ the same variables, namely $m_{\ell \ell }$, $p_{\text {T}} ^{\ell \ell }$, $\Delta \phi _{\ell \ell }$ and $m_\mathrm{T}$, which provide the best discrimination between signal hypotheses and backgrounds, also in the presence of one jet in the final state. All background components are used in the trainings. In total, five BDT$_2$ trainings are performed for the alternative spin hypotheses (one for the spin-2 UC scenario and two for each of the two spin-2 non-UC hypotheses corresponding to the different $p_{\text {T}} ^{X}$ selections), plus one training of BDT$_0$ for the SM Higgs boson hypothesis.

For the spin-0 fixed hypothesis test and *HWW* tensor structure studies, the first discriminant, BDT$_0$, is the same as the one used for the spin-2 analysis, trained to disentangle the SM hypothesis from the background. A second BDT discriminant, $\text{ BDT }_{bad hbox}$, is obtained by training the SM signal versus the alternative signal sample (the pure CP-even or CP-odd BSM hypotheses), and then applied to all CP-mixing fractions. No background component is involved in this case. The variables used for the $\text{ BDT }_{bad hbox}$ trainings are $m_{\ell \ell }$, $\Delta \phi _{\ell \ell }$, $p_{\text {T}} ^{\ell \ell }$  and the missing transverse momentum for the CP-even analysis and $m_{\ell \ell }$, $\Delta \phi _{\ell \ell }$, $E_{\ell \ell \nu \nu }$  and $\Delta p_\mathrm{T}$ for the CP-odd analysis. The training strategy is different from the one used in the spin-2 analysis because, while the spin-2 signal is very similar to the background, the spin-0 signals are all similar to each other, while being different from the main background components. Therefore, in the latter case, training the signal hypotheses against each other improves the sensitivity. The resulting BDT variable is afterwards used in binned likelihood fits to test the data for compatibility with the presence of a SM or BSM Higgs boson.

Several sources of systematic uncertainty are considered, both from experimental and theoretical sources, and are described in detail in Ref. [8]. The correlations induced among the different background sources by the presence of other processes in the control regions are fully taken into account in the statistical procedure. The most important systematic uncertainties are found to be those related to the modelling of the *WW* background, to the estimate of the *W*+jets background (originating from the data-driven method employed) and, for the spin-2 results in particular, to the $Z\rightarrow \tau \tau$ modelling.

### Spin and parity analysis in the $H \rightarrow ZZ^{*} \rightarrow 4 \ell$ channel 

The reconstruction of physics objects and event selection used for the $H \rightarrow ZZ^{*} \rightarrow 4 \ell$ analysis is identical to the one presented in Ref. [12]. The main improvement with respect to the previous ATLAS publication of Ref. [4] is the introduction of a BDT discriminant designed to optimise the separation between the signal and the most relevant background process.

Events containing four reconstructed leptons (electrons or muons) in the final state are selected using single-lepton and dilepton triggers. The selected events are classified according to their final state: $4\mu ,~2e2\mu ,~2\mu 2e$ and 4*e*, where for the decay modes $2e2\mu$ and $2\mu 2e$ the first pair is defined to be the one with the dilepton mass closest to the *Z* boson mass. Each muon (electron) must satisfy $p_{\text {T}} > 6$ GeV ($p_{\text {T}} > 7$ GeV) and be measured in the pseudorapidity range $|\eta |<$ 2.7 ($|\eta |<$ 2.47). Higgs boson candidates are formed by selecting two same-flavour, opposite-charge lepton pairs in an event. The lepton with the highest $p_{\text {T}}$ in the quadruplet must have $p_{\text {T}} >20$ GeV, and the leptons with the second- and third-highest $p_{\text {T}}$ must have $p_{\text {T}} >15$ GeV and $p_{\text {T}} >$10 GeV, respectively. The lepton pair with the mass closest to the *Z* boson mass is referred to as the leading lepton pair and its invariant mass as $m_{12}$. The requirement $50~\text {GeV}< m_{12} < 106~\text {GeV}$ is applied. The other lepton pair is chosen from the remaining leptons as the pair closest in mass to the *Z* boson. Its mass, denoted hereafter by $m_{34}$, must satisfy $12~\text {GeV}< m_{34} < 115 ~\text {GeV}$. Further requirements are made on the impact parameters of the leptons relative to the interaction vertex and their isolation in both the tracker and calorimeter.

The main background process affecting the selection of $H \rightarrow ZZ^{*} \rightarrow 4 \ell$ events is the non-resonant production of $ZZ^*$ pairs. This background has the same final state as the signal events and hereafter is referred to as the irreducible background. It is estimated from simulation and normalised to the expected SM cross section calculated at NLO [37, 38]. The reducible sources of background come from *Z*+jets and $t\bar{t}$ processes, where additional leptons arise due to misidentified jets or heavy-flavour decays. The rate and composition of the reducible backgrounds are evaluated using data-driven techniques, separately for the two final states with sub-leading muons $\ell \ell +\mu \mu$ and those with sub-leading electrons $\ell \ell +ee$.

Only events with an invariant mass of the four-lepton system, denoted by $m_{4\ell }$, satisfying the signal region definition 115 GeV $<m_{4\ell }<$130 GeV are selected. The expected signal and background yields in the signal region and the observed events in data are reported in Table 3.

**Table 3:** Expected signal, background and total yields, including their total uncertainties, and observed events in data, in the 115 GeV $< m_{4\ell } <$ 130 GeV signal region. The number of expected signal events is given for a SM Higgs boson mass of 125.5 GeV

	SM Signal	$ZZ^*$	$t\bar{t}, Z+\mathrm{jets}$	Total expected	Observed
$\sqrt{s} =7$ $\text {TeV}$					
$4\mu$	1.02 $\pm$ 0.10	0.65 $\pm$ 0.03	0.14 $\pm$ 0.06	1.81 $\pm$ 0.12	3
$2\mu 2e$	0.47 $\pm$ 0.05	0.29 $\pm$ 0.02	0.53 $\pm$ 0.12	1.29 $\pm$ 0.13	1
$2e 2\mu$	0.64 $\pm$ 0.06	0.45 $\pm$ 0.02	0.13 $\pm$ 0.05	1.22 $\pm$ 0.08	2
4*e*	0.45 $\pm$ 0.04	0.26 $\pm$ 0.02	0.59 $\pm$ 0.12	1.30 $\pm$ 0.13	2
Total	2.58 $\pm$ 0.25	1.65 $\pm$ 0.09	1.39 $\pm$ 0.26	5.62 $\pm$ 0.37	8
$\sqrt{s} =8$ $\text {TeV}$					
$4\mu$	5.81 $\pm$ 0.58	3.36 $\pm$ 0.17	0.97 $\pm$ 0.18	10.14 $\pm$ 0.63	13
$2\mu 2e$	3.00 $\pm$ 0.30	1.59 $\pm$ 0.10	0.52 $\pm$ 0.12	5.11 $\pm$ 0.34	8
$2e 2\mu$	3.72 $\pm$ 0.37	2.33 $\pm$ 0.11	0.84 $\pm$ 0.14	6.89 $\pm$ 0.41	9
4*e*	2.91 $\pm$ 0.29	1.44 $\pm$ 0.09	0.52 $\pm$ 0.11	4.87 $\pm$ 0.32	7
Total	15.4 $\pm$ 1.5	8.72 $\pm$ 0.47	2.85 $\pm$ 0.39	27.0 $\pm$ 1.6	37

**Fig. 3:**
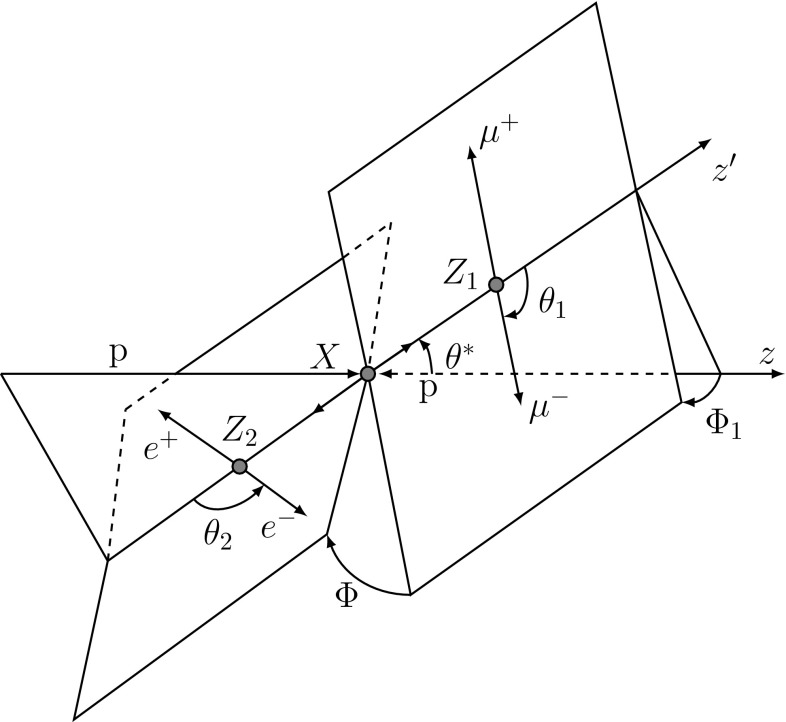
Definitions of the angular observables sensitive to the spin and parity of the resonance in the $X \rightarrow ZZ^{*}\rightarrow 4\ell$ decay

The choice of production and decay angles used in this analysis is presented in Fig. 3, where the following definitions are used:

- $\theta _1$ and $\theta _2$ are defined as the angles between final-state leptons with negative charge and the direction of flight of their respective *Z* bosons, in the four-lepton rest frame;
- $\Phi$ is the angle between the decay planes of two lepton pairs (matched to the two *Z* boson decays) expressed in the four-lepton rest frame;
- $\Phi _1$ is the angle between the decay plane of the leading lepton pair and a plane defined by the $Z_1$ momentum (the *Z* boson associated with the leading lepton pair) in the four-lepton rest frame and the positive direction of the collision axis;
- $\theta ^{*}$ is the production angle of the $Z_1$ defined in the four-lepton rest frame.

**Fig. 4:**
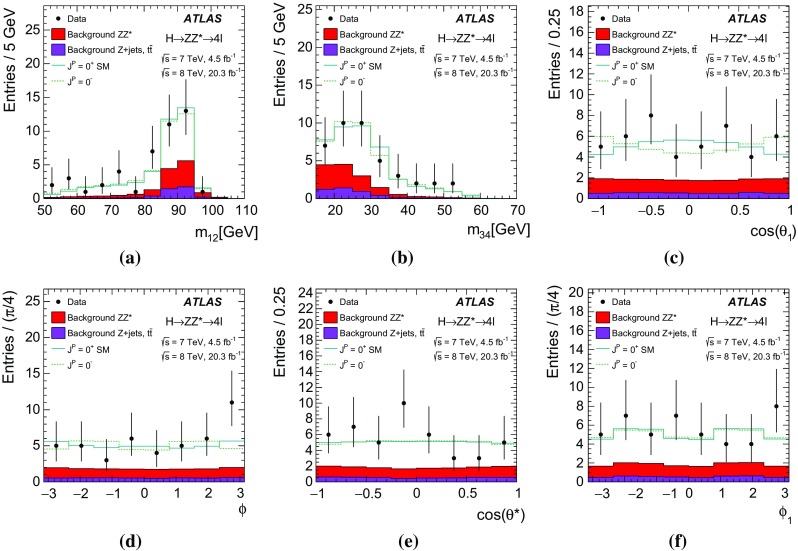
Distributions of some of the final-state observables sensitive to the spin and parity of the resonance in the $H\rightarrow ZZ^{*}\rightarrow 4\ell$ signal region $115~\text {GeV}<m_{4\ell }< 130~\text {GeV}$ for data (points with errors), backgrounds (*filled histograms*) and predictions for two spin hypotheses (SM *solid line* and *alternatives dashed lines*). **a**–**c** Invariant masses $m_{12}$ , $m_{34}$ and decay $\cos \theta _1$, respectively; **d**–**f** $\Phi$, $\cos \theta ^*$ and $\Phi _1$, respectively

The final-state observables sensitive to the spin and parity of a boson decaying to $ZZ^{*}\rightarrow 4\ell$ are the two production angles $\theta ^{*}$ and $\Phi _1$ and the three decay angles $\Phi$, $\theta _1$ and $\theta _2$. In the case of a spin-0 boson, the differential production cross section does not depend on the production variables $\cos (\theta ^{*})$ and $\Phi _1$. It should be noted that, as the Higgs boson mass is below $2m_Z$, the shapes of the mass distributions of the intermediate *Z* bosons, $m_{12}$ and $m_{34}$, are sensitive to the spin and parity of the resonance. In Fig. 4 the distributions of the final-state observables sensitive to the spin and parity of the decaying resonance are presented. The distributions are shown for the SM $J^P=0^+$ and $J^P=0^-$ simulated events, as well as for $ZZ^{*}$ production and reducible backgrounds in the signal region $115~\text {GeV}<m_{4\ell }< 130~\text {GeV}$. The events observed in data are superimposed on each plot.

Two approaches were pursued to develop the discriminants used to distinguish between different spin and parity hypotheses. The first uses the theoretical differential decay rate for the final-state observables sensitive to parity to construct a matrix-element-based likelihood ratio analysis ($J^P$-MELA). The second approach is based on a BDT.

For the $J^P$-MELA approach [3, 9], the probability of observing an event with given kinematics can be calculated. This probability is corrected for detector acceptance and analysis selection, which are obtained from the simulated signal MC samples. The full pdf also includes a term for incorrect pairing of the leptons in the $4\mu$ and 4*e* channels. For a given pair of spin-parity hypotheses under test, the final discriminant is defined as the ratio of the pdf for a given hypothesis to the sum of the pdfs for both hypotheses.

For the BDT approach, a $J^P$ discriminant is formed for each pair of spin-parity states to be tested, by training a BDT on the variables of simulated signal events which fall in the signal mass window $115~\text {GeV}<m_{4\ell } <130~\text {GeV}$. For the $0^+$ versus $0^-$ test, only the parity-sensitive observables $\Phi$, $\theta _1$, $\theta _ 2$, $m_{12}$ and $m_{34}$ are used in the BDT training. For the spin-2 test, the production angles $\theta ^*$ and $\Phi _1$ are also included.

**Fig. 5:**
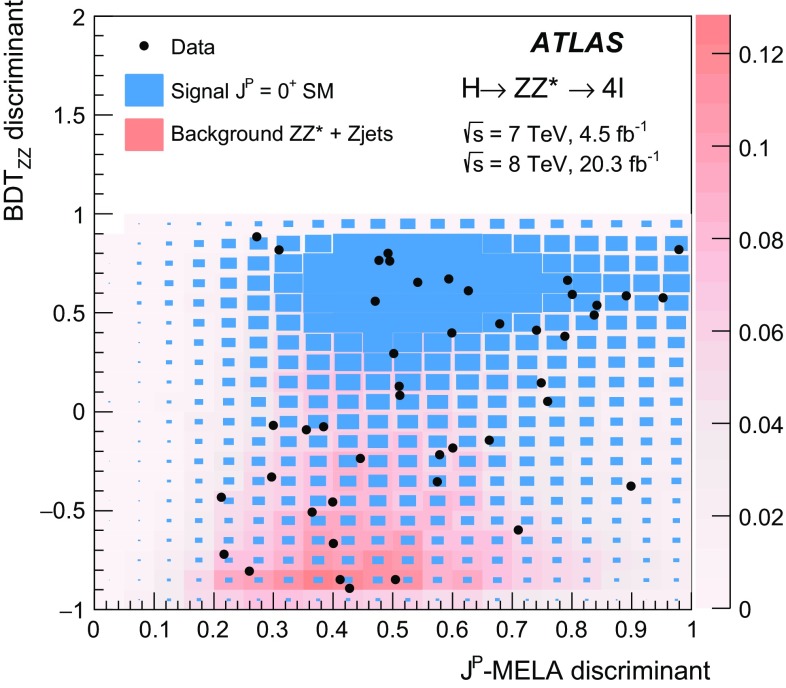
The distributions of the discriminant BDT$_{ZZ}$ versus the $J^P$-MELA discriminant for the SM $J^P=0^+$ Higgs boson and for the backgrounds in the $H \rightarrow ZZ^{*} \rightarrow 4 \ell$ signal region $115~\text {GeV}\ <m_{4\ell } <130~\text {GeV}$

**Fig. 6:**
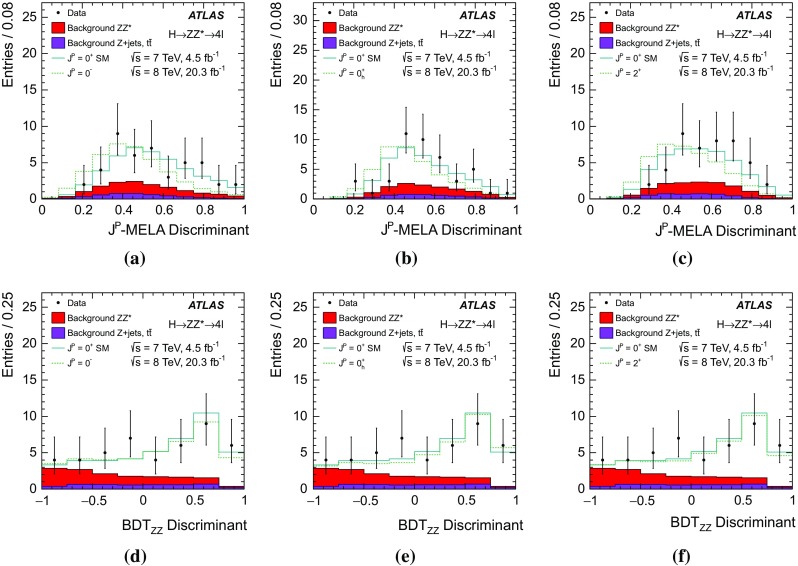
Distributions of the $J^P$-MELA and of the $\mathrm{BDT}_{ZZ}$ discriminants in the $H\rightarrow ZZ^{*}\rightarrow 4\ell$ signal region $115~\text {GeV}<m_{4\ell }< 130~\text {GeV}$ for the data (points with errors), the backgrounds (*filled histograms*), and for predictions for several spin and parity hypotheses. The SM hypothesis is shown by the *solid line* while the alternative hypotheses are shown by the *dashed lines*. The signal distributions are normalised to the signal strength fitted in data. **a**–**c** $J^P$-MELA discriminants for $0^+$ SM vs $0^-$, $0^+$ SM vs $0^+_h$ and $0^+$ SM vs $2^+$, respectively; **d**–**f** $\mathrm{BDT}_{ZZ}$ discriminant for $0^+$ SM vs $0^-$, $0^+$ SM vs $0^+_h$ and $0^+$ SM vs $2^+$, respectively

Both analyses are complemented with a BDT discriminant designed to separate the signal from the $ZZ^*$ background. These discriminants are hereafter referred to as BDT$_{ZZ}$. For the $J^P$-MELA analysis, the BDT$_{ZZ}$ discriminant is fully equivalent to the one described in Refs. [12, 18]. For the BDT analysis the discriminating variables used for the background BDT$_{ZZ}$ are the invariant mass, pseudorapidity, and transverse momentum of the four-lepton system, and a matrix-element-based kinematic discriminant $K_D$ defined in Ref. [16]. The results from both methods are obtained from likelihood fits to the two-dimensional distributions of the background BDTs and of the spin- and parity-sensitive discriminants. In this way, the small correlation between these variables are taken into account in the analyses. The distribution of the background discriminant BDT$_{ZZ}$ versus the $J^P$-MELA discriminant is presented in Fig. 5 for the SM $J^P=0^+$ signal, the backgrounds, and the data. The projections of this distribution on the $J^P$-MELA and the BDT$_{ZZ}$ variables, for different signal hypotheses, the backgrounds, and the data, are shown in Fig. 6. In this paper, only results based on the $J^P$-MELA approach are reported. The BDT approach was used as a cross-check and produced compatible results.

Two general types of systematic effects impact the analyses using fixed spin and parity hypotheses: uncertainties on discriminant shapes due to experimental effects, and uncertainties on background normalisations from theory uncertainties and data-driven background estimates. The systematic uncertainties on the shape are included in the analysis by creating discriminant shapes corresponding to variations of one standard deviation in the associated sources of systematic uncertainty. The systematic uncertainties on the normalisation are included as additional nuisance parameters in the likelihood.

The list of sources of systematic uncertainty common to all ATLAS $H \rightarrow ZZ^{*} \rightarrow 4 \ell$ analyses is presented in Ref. [18]. The relative impact of these sources on the final separation for all tested hypotheses is evaluated and sources affecting the final separation (given in Sect. 5.5) by less than $\pm 0.5\,\%$ are neglected.

The main sources of systematic uncertainties are related to the experimental error on the Higgs boson mass, the modelling of the irreducible $ZZ^{*}$ background, the uncertainty on the integrated luminosity and the experimental uncertainties on the electron and muon reconstruction. The uncertainty on the Higgs boson mass affects the final result since it impacts the shapes of the $m_{12}$, $m_{34}$, $\cos \theta _{1}$ and $\cos \theta _{2}$ variables. For the $J^P$-MELA method, the uncertainty on the estimate of the fraction of $4\mu$ and 4*e* candidates with an incorrect pairing of leptons is also considered. This uncertainty is derived by comparing the corresponding prediction obtained from the Powheg and JHU MC generators for the SM hypothesis. A variation of $\pm 10\,\%$ of the incorrect pairing fraction is applied to all spin and parity hypotheses.

**Table 4:** Relative impact of the main systematic uncertainties on the expected separation (expressed in terms of numbers of standard deviations) between the SM $J^P=0^+$ and $J^P=0^-$ hypotheses for the $H \rightarrow ZZ^{*} \rightarrow 4 \ell$ $J^P$-MELA analysis

Source of the systematic uncertainty	Relative impact (%)
Higgs boson mass experimental uncertainty	$\pm 2$
$ZZ^{*}$ pdf	$\pm 0.8$
Muon momentum scale	$\pm 0.7$
$Zbb\rightarrow \ell \ell \mu \mu$ normalisation	$\pm 0.6$
$ZZ^{*}$ scale	$\pm 0.6$
Luminosity	$\pm 0.6$
$e/\gamma$ resolution model (sampling term)	$\pm 0.5$
$e/\gamma$ resolution model (constant term)	$\pm 0.5$
$Z\rightarrow \ell \ell ee$ normalisation	$\pm 0.5$
Fraction of wrongly paired $4\ell$ candidates	$\pm 0.4$

**Fig. 7:**
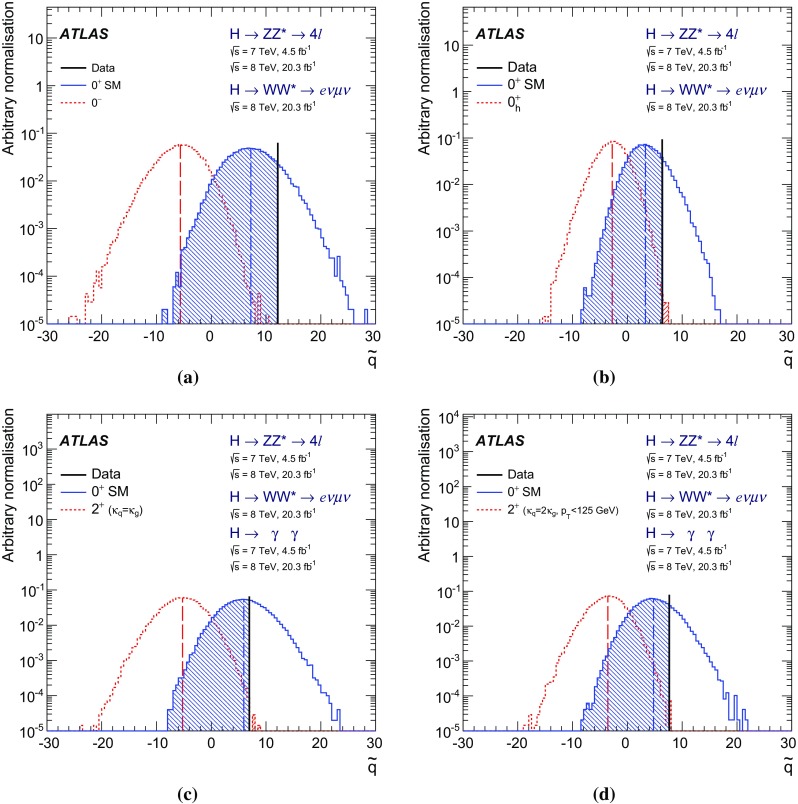
Examples of distributions of the test statistic $\tilde{q}$ defined in Sect. 5.1, for the combination of decay channels. **a** $0^+$ versus $0^-$; **b** $0^+$ versus $0^+ _h$; **c** $0^+$ versus the spin-2 model with universal couplings ($\kappa _{q} = \kappa _{g}$); **d** $0^+$ versus the spin-2 model with $\kappa _{q} = 2 \kappa _{g}$ and the $p_{\text {T}}$ selection at 125 GeV. The observed values are indicated by the *vertical solid line* and the expected medians by the *dashed lines*. The *shaded areas* correspond to the integrals of the expected distributions used to compute the *p*-values for the rejection of each hypothesis

The influence of the main systematic uncertainties on the separation between the SM $J^P=0^+$ and $J^P=0^-$ hypotheses for the $J^P$-MELA analysis is presented in Table 4. The total relative impact of all systematic uncertainties on the separation between the hypotheses (expressed in terms of numbers of standard deviations) is estimated to be about $\pm 3\,\%$.

### Individual and combined results

The distributions of discriminant variables in data agree with the SM predictions for all three channels, and exclusion ranges for alternative spin hypotheses are derived. Some examples of distributions of the test statistic $\tilde{q}$ (defined in Sect. 5.1) used to derive the results are presented in Fig. 7. In this figure, the observed value is indicated by the vertical solid line and the expected medians by the dashed lines. The shaded areas correspond to the integrals of the expected distributions used to compute the *p*-values for the rejection of each hypothesis. The signal strengths per decay channel and per centre-of-mass energy are treated as independent parameters in each fit. Their values are compatible with the SM predictions.

The results obtained from the fit to the data, expressed in terms of *p*-values for different tested hypotheses and observed $\mathrm{CL}_\mathrm{s}$ for the alternative hypotheses, are summarised in Tables 5 and 6. As shown in Table 5, the sensitivity to reject alternative hypotheses is driven by the $H \rightarrow ZZ^{*} \rightarrow 4 \ell$ and the $H \rightarrow WW^{*} \rightarrow e \nu \mu \nu$ channels. The $H \rightarrow \gamma \gamma$ channel has sizeable sensitivity only to spin-2 models where the $p_{\text {T}} ^{X}<125 \text {GeV}$ selection is not applied. In all cases the data prefer the SM hypothesis to the alternative models, with the exception of some of the spin-2 models for the $H \rightarrow \gamma \gamma$ channel. In this case both hypotheses have similar observed *p*-values, but neither of the two is below 10 %.

As summarised in Table 6, the *p*-values of the combined results for the three channels show good agreement between the data and the SM hypothesis for all performed tests. All tested alternative hypotheses are rejected at a more than 99.9 % confidence level (CL) in favour of the SM hypothesis.

**Table 5:** Expected and observed *p*-values for different spin-parity hypotheses, for each of the three channels $H \rightarrow \gamma \gamma$, $H \rightarrow ZZ^{*} \rightarrow 4 \ell$, and $H \rightarrow WW^{*} \rightarrow e \nu \mu \nu$. The observed $\mathrm{CL}_\mathrm{s}$ for the alternative hypotheses are reported in the last column. The expected and observed *p*-values and the observed $\mathrm{CL}_\mathrm{s}$ are defined in Sect. 5.5 and the alternative hypotheses are those described in Sect. 3

Tested hypothesis	$p^\mathrm{alt}_{\mathrm{exp},\mu =1}$	$p^\mathrm{alt}_{\mathrm{exp},\mu =\hat{\mu }}$	$p^\mathrm{SM}_\mathrm{obs}$	$p^\mathrm{alt}_\mathrm{obs}$	Obs. $\mathrm{CL}_\mathrm{s}$ (%)
$$H \rightarrow \gamma \gamma $$					
$2^+ (\kappa _q = \kappa _g)$	0.13	$7.5 \times 10^{-2}$	0.13	0.34	39
$2^+(\kappa _q =0;\; p_{\text {T}} <300 \text {GeV})$	$4.3 \times 10^{-4}$	${<}3.1 \times 10^{-5}$	0.16	2.9$\times 10^{-4}$	3.5$\times 10^{-2}$
$2^+(\kappa _q =0;\; p_{\text {T}} <125 \text {GeV})$	$9.4 \times 10^{-2}$	5.6$\times 10^{-2}$	0.23	0.20	26
$2^+(\kappa _q =2 \kappa _g ;\; p_{\text {T}} <300\text {GeV})$	$9.1 \times 10^{-4}$	${<}3.1 \times 10^{-5}$	0.16	8.6$\times 10^{-4}$	0.10
$2^+(\kappa _q =2 \kappa _g ;\; p_{\text {T}} <125\text {GeV})$	0.27	0.24	0.20	0.54	68
$$H \rightarrow WW^{*} \rightarrow e \nu \mu \nu $$					
$0^+_h$	0.31	0.29	0.91	2.7$\times 10^{-2}$	29
$0^-$	6.4$\times 10^{-2}$	3.2$\times 10^{-2}$	0.65	1.2$\times 10^{-2}$	3.5
$2^+(\kappa _q = \kappa _g)$	6.4$\times 10^{-2}$	3.3$\times 10^{-2}$	0.25	0.12	16
$2^+(\kappa _q =0;\; p_{\text {T}} <300\text {GeV})$	1.5$\times 10^{-2}$	4.0$\times 10^{-3}$	0.55	3.0$\times 10^{-3}$	0.6
$2^+(\kappa _q =0;\; p_{\text {T}} <125\text {GeV})$	5.6$\times 10^{-2}$	2.9$\times 10^{-2}$	0.42	4.4$\times 10^{-2}$	7.5
$2^+(\kappa _q =2 \kappa _g ;\; p_{\text {T}} <300\text {GeV})$	1.5$\times 10^{-2}$	4.0$\times 10^{-3}$	0.52	3.0$\times 10^{-3}$	0.7
$2^+(\kappa _q =2 \kappa _g ;\; p_{\text {T}} <125\text {GeV})$	4.4$\times 10^{-2}$	2.2$\times 10^{-2}$	0.69	7.0$\times 10^{-3}$	2.2
$$H \rightarrow ZZ^{*} \rightarrow 4 \ell $$					
$0^+_h$	$3.2 \times 10^{-2}$	$5.2 \times 10^{-3}$	0.80	$3.6 \times 10^{-4}$	0.18
$0^-$	$8.0 \times 10^{-3}$	$3.6 \times 10^{-4}$	0.88	$1.2 \times 10^{-5}$	1.0$\times 10^{-2}$
$2^+(\kappa _q = \kappa _g)$	$3.3 \times 10^{-2}$	$5.7 \times 10^{-4}$	0.91	$3.6 \times 10^{-5}$	4.0$\times 10^{-2}$
$2^+(\kappa _q =0;\; p_{\text {T}} <300\text {GeV})$	$3.9 \times 10^{-2}$	$9.0 \times 10^{-3}$	0.95	$2.7 \times 10^{-5}$	5.4$\times 10^{-2}$
$2^+(\kappa _q =0;\; p_{\text {T}} <125\text {GeV})$	$4.6 \times 10^{-2}$	$1.1 \times 10^{-2}$	0.93	$3.0 \times 10^{-5}$	4.3$\times 10^{-2}$
$2^+(\kappa _q =2 \kappa _g ;\; p_{\text {T}} <300\text {GeV})$	$4.6 \times 10^{-2}$	$1.1 \times 10^{-2}$	0.66	$3.3 \times 10^{-3}$	0.97
$2^+(\kappa _q =2 \kappa _g ;\; p_{\text {T}} <125\text {GeV})$	$5.0 \times 10^{-2}$	$1.3 \times 10^{-2}$	0.88	$3.2 \times 10^{-4}$	0.27

**Table 6:** Expected and observed *p*-values for different spin-parity hypotheses, for the combination of the three channels: $H \rightarrow \gamma \gamma$ , $H \rightarrow ZZ^{*} \rightarrow 4 \ell$ and $H \rightarrow WW^{*} \rightarrow e \nu \mu \nu$. The observed $\mathrm{CL}_\mathrm{s}$ for the alternative hypothesis is reported in the last column. The expected and observed *p*-values and the observed $\mathrm{CL}_\mathrm{s}$ are defined in Sect. 5.5. The definitions of alternative hypotheses are given in Sect. 3

Tested hypothesis	$p^\mathrm{alt}_{\mathrm{exp},\mu =1}$	$p^\mathrm{alt}_{\mathrm{exp},\mu =\hat{\mu }}$	$p^\mathrm{SM}_\mathrm{obs}$	$p^\mathrm{alt}_\mathrm{obs}$	Obs. $\mathrm{CL}_\mathrm{s}$ (%)
$0^+_h$	$2.5 \times 10^{-2}$	$4.7 \times 10^{-3}$	0.85	$7.1 \times 10^{-5}$	$4.7 \times 10^{-2}$
$0^-$	$1.8 \times 10^{-3}$	$1.3 \times 10^{-4}$	0.88	${<}3.1 \times 10^{-5}$	${<}2.6 \times 10^{-2}$
$2^+ (\kappa _q = \kappa _g)$	$4.3 \times 10^{-3}$	$2.9 \times 10^{-4}$	0.61	$4.3 \times 10^{-5}$	$1.1 \times 10^{-2}$
$2^+ (\kappa _q =0;\; p_{\text {T}} <300\text {GeV})$	${<}3.1 \times 10^{-5}$	${<}3.1 \times 10^{-5}$	0.52	${<}3.1 \times 10^{-5}$	${<}6.5 \times 10^{-3}$
$2^+ (\kappa _q =0;\;p_{\text {T}} <125\text {GeV})$	$3.4 \times 10^{-3}$	$3.9 \times 10^{-4}$	0.71	$4.3 \times 10^{-5}$	$1.5 \times 10^{-2}$
$2^+ (\kappa _q =2 \kappa _g ;\; p_{\text {T}} <300\text {GeV})$	${<}3.1 \times 10^{-5}$	${<}3.1 \times 10^{-5}$	0.28	${<}3.1 \times 10^{-5}$	${<}4.3 \times 10^{-3}$
$2^+ (\kappa _q =2 \kappa _g;\; p_{\text {T}} <125\text {GeV})$	$7.8 \times 10^{-3}$	$1.2 \times 10^{-3}$	0.80	$7.3 \times 10^{-5}$	$3.7 \times 10^{-2}$

## Study of CP-mixing and of the *HVV* interaction tensor structure 

Following the discussion in Sect. 3, measurements of the *HVV* interaction tensor couplings $\kappa _\mathrm{SM}$, $\kappa _{AVV}$, $\kappa _{HVV}$ and of the mixing angle $\alpha$ are performed. The measurements consist of fitting the ratios of couplings $(\tilde{\kappa } _{AVV}/\kappa _\mathrm{SM}) \cdot \tan \alpha$ and $\tilde{\kappa } _{HVV}/\kappa _\mathrm{SM}$ to the discriminant observables for the $H \rightarrow WW^{*} \rightarrow e \nu \mu \nu$ and $H \rightarrow ZZ^{*} \rightarrow 4 \ell$ processes and in their combination. In the fitting procedure only one ratio of couplings $(\tilde{\kappa } _{AVV}/\kappa _\mathrm{SM}) \cdot \tan \alpha$ or $\tilde{\kappa } _{HVV}/\kappa _\mathrm{SM}$ is considered at a time, while the other one is assumed to be absent.

### Statistical treatment

The measurement of the tensor structure of the *HVV* interaction is based on a profiled likelihood [31, 32] that contains the discriminant observables sensitive to the EFT couplings. The signal rates in the different channels and for different centre-of-mass energies are treated as independent parameters. Therefore, the global signal normalisation is not used to constrain the EFT couplings. The ratios of the BSM to SM couplings, $\tilde{\kappa }_{HVV}/\kappa _\mathrm{SM}$ and $(\tilde{\kappa }_{AVV}/\kappa _\mathrm{SM})\cdot \tan {\alpha }$, are each separately fit to the discriminant observables in data. The test statistic used to derive the confidence intervals on the parameters of interest is $q' = -2 \ln (\lambda )$, where $\lambda$ is the profiled likelihood [31, 32]. The results presented in the following rely on the asymptotic approximation [31, 32] for the test statistic. This approximation was cross-checked with Monte Carlo ensemble tests that confirm its validity in the range of the parameters for which the 95 % CL limits are derived.

### Tensor structure analyses in the $H \rightarrow WW^{*} \rightarrow e \nu \mu \nu$ channel

The $H \rightarrow WW^{*} \rightarrow e \nu \mu \nu$ analysis used to study the spin-0 tensor structure is already described in Sect. 5.3 and detailed in Ref. [8]. Only the 0-jet category is considered and the BDT$_0$ and $\text{ BDT }_{bad hbox}$ are used as discriminant variables in the likelihood defined to measure the spin-0 tensor structure couplings. The only difference with respect to the spin hypothesis test is that, in this analysis, the BSM spin-0 couplings are treated as continuous variables in the test statistic.

### Tensor structure analyses in the $H \rightarrow ZZ^{*} \rightarrow 4 \ell$ channel 

To allow for a cross-check and validation of the obtained results, two different fitting methods based on the analytical calculation of the leading-order matrix element of the $H \rightarrow ZZ^{*} \rightarrow 4 \ell$ process are used.

The method of the matrix-element-observable fit is based on modelling the distributions of the final-state observables in each bin of coupling ratios using Monte Carlo simulation. Using the Lagrangian defined in Eq. (1), which is linear in the coupling constants $\kappa _\mathrm{SM}$, $\kappa _{HVV}$ and $\kappa _{AVV}$, the differential cross section at each point in the phase space can be expressed as a term corresponding to the SM amplitude, plus two additional terms, linear and quadratic in the coupling constants. In this way it is possible to define two observables for each coupling, the so-called first- and second-order optimal observables, upon which the amplitude depends at each point of the phase space. For each event, they contain the full kinematic information about the couplings, which can thus be extracted from a fit to their shapes. More details of the method can be found in Refs. [39–42].

The observables sensitive to the presence and structure of $\kappa _\mathrm{SM}$, $\kappa _{HVV}$ and $\kappa _{AVV}$ considered in the current analysis are defined as follows:

10 $$\begin{aligned}&O_1(\kappa _{HVV}) = \frac{2\mathfrak {R}[\mathrm{ME}(\kappa _\mathrm{SM} \ne 0;\; \kappa _{HVV}, \kappa _{AVV} = 0;\; \alpha =0 )^* \cdot \mathrm{ME}( \kappa _{HVV} \ne 0;\; \kappa _\mathrm{SM}, \kappa _{AVV}=0;\; \alpha =0)] }{|\mathrm{ME}(\kappa _\mathrm{SM} \ne 0;\; \kappa _{HVV}, \kappa _{AVV} = 0;\; \alpha =0)|^2}, \nonumber \\&O_2(\kappa _{HVV}) = \frac{|\mathrm{ME}(\kappa _{HVV} \ne 0;\; \kappa _\mathrm{SM}, \kappa _{AVV}=0;\; \alpha =0 )|^2}{|\mathrm{ME}(\kappa _\mathrm{SM} \ne 0;\; \kappa _{HVV}, \kappa _{AVV} = 0;\; \alpha =0)|^2}, \nonumber \\&O_1(\kappa _{AVV},\alpha ) = \frac{2\mathfrak {R}[\mathrm{ME}(\kappa _\mathrm{SM} \ne 0;\; \kappa _{HVV}, \kappa _{AVV} = 0;\; \alpha =0 )^* \cdot \mathrm{ME}( \kappa _{AVV} \ne 0;\; \kappa _\mathrm{SM}, \kappa _{HVV}=0;\; \alpha =\pi /2)] }{|\mathrm{ME}(\kappa _\mathrm{SM} \ne 0;\; \kappa _{HVV}, \kappa _{AVV} = 0;\; \alpha =0)|^2}, \nonumber \\&O_2(\kappa _{AVV},\alpha ) =\frac{|\mathrm{ME}( \kappa _{AVV} \ne 0;\; \kappa _\mathrm{SM}, \kappa _{HVV}=0;\; \alpha =\pi /2)|^2}{|\mathrm{ME}(\kappa _\mathrm{SM} \ne 0;\; \kappa _{HVV}, \kappa _{AVV} = 0;\; \alpha =0)|^2}. \end{aligned}$$

Here $\mathrm{ME}(\kappa _\mathrm{SM},\kappa _{HVV},\kappa _{AVV},\alpha )$ denotes the leading-order matrix element of the $H \rightarrow ZZ^{*} \rightarrow 4 \ell$ process. These definitions correspond to the first- and second-order optimal observables for a BSM amplitude with a three-component structure.

The observables $O_{1,2}(\kappa _{HVV})$ and $O_{1,2}(\kappa _{AVV},\alpha )$ are used for the $\tilde{\kappa } _{HVV}/\kappa _\mathrm{SM}$ and $(\tilde{\kappa } _{AVV}/\kappa _\mathrm{SM}) \cdot \tan \alpha$ individual fits respectively. In order to suppress the $ZZ^{*}$ background, a kinematic BDT discriminant similar to those described in Sect. 5.4 is used as an additional observable in all fits. The BDT training is performed independently for each final state using observables with small sensitivity to parity: $\eta _{4\ell }$, $p_{\mathrm{T},4\ell }$, $m_{4\ell }$, $\cos (\theta ^*)$ and $\Phi _1$. This BDT discriminant is denoted hereafter by $\mathrm{BDT(}ZZ\mathrm {)}$.

**Fig. 8:**
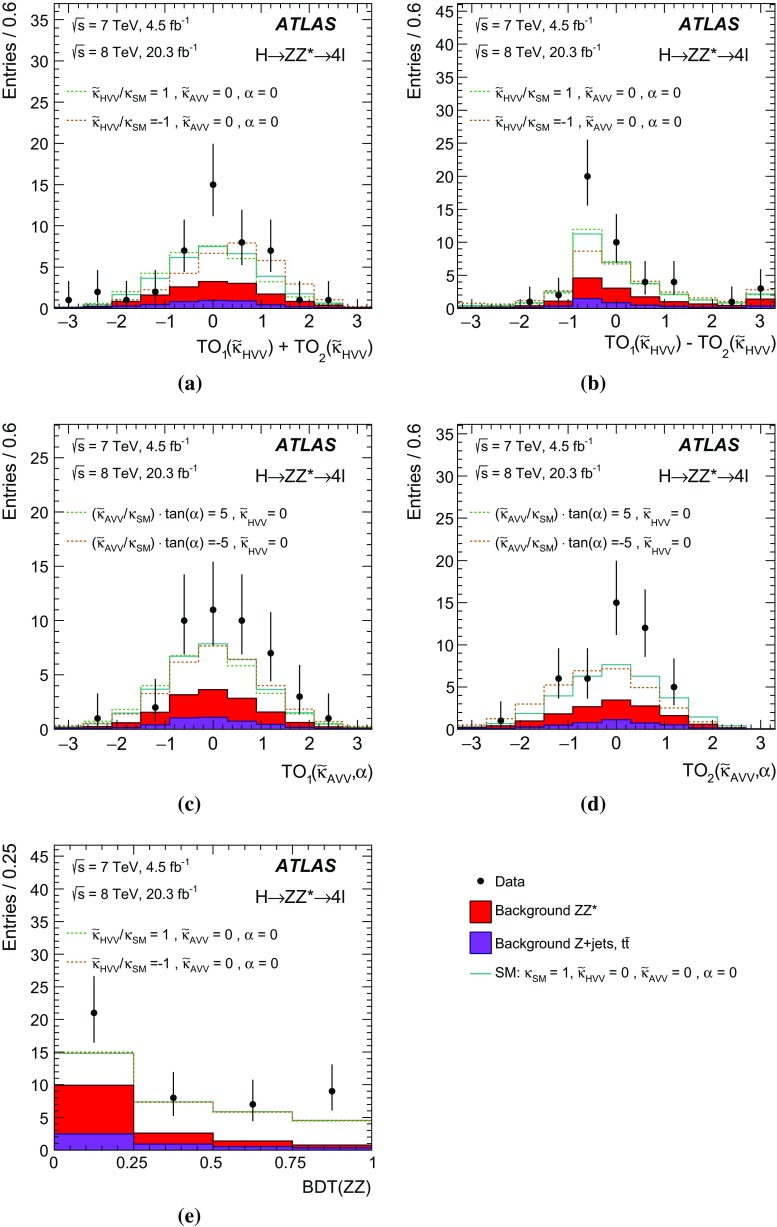
Distributions of the observables used in the matrix-element-observable fit. **a** $TO_1(\tilde{\kappa }_{HVV}) + TO_2(\tilde{\kappa }_{HVV})$, **b** $TO_1(\tilde{\kappa }_{HVV}) - TO_2(\tilde{\kappa }_{HVV})$, for the Monte Carlo signal generated with $(\tilde{\kappa } _{HVV}/\kappa _\mathrm{SM} =0,\pm 1; \kappa _{AVV} =0)$. **c** $TO_{1}(\tilde{\kappa }_{AVV} , \alpha )$, **d** $TO_{2}(\tilde{\kappa }_{AVV} , \alpha )$ for the Monte Carlo signal generated with $((\tilde{\kappa }_{AVV}/\kappa _\mathrm{SM}) \cdot \tan \alpha =0,\pm 5; \kappa _{HVV} =0)$. **e** $\mathrm{BDT(}ZZ\mathrm {)}$ for the Monte Carlo signal generated with $(\tilde{\kappa } _{HVV}/\kappa _\mathrm{SM} =0,\pm 1; \kappa _{AVV} =0)$. The expected background contributions are shown as *filled histograms* on each plot

To simplify their use in the analysis, all observables defined in Eq. (10) undergo a pdf transformation such that each observable becomes normally distributed in the Standard Model case. These transformed observables are referred to hereafter as $TO_{1,2}(\kappa _{HVV})$ and $TO_{1,2}(\kappa _{AVV},\alpha )$ respectively. The distributions of transformed observables for the Monte Carlo signal samples generated with $(\tilde{\kappa } _{HVV}/\kappa _\mathrm{SM} =0,\pm 1; \tilde{\kappa } _{AVV} =0)$ and $((\tilde{\kappa } _{AVV}/\kappa _\mathrm{SM}) \cdot \tan \alpha =0,\pm 5; \tilde{\kappa } _{HVV} =0)$ are shown in Fig. 8.

**Table 7:** Fitted values of $\tilde{\kappa }_{HVV}/\kappa _\mathrm{SM}$ and $(\tilde{\kappa }_{AVV}/\kappa _\mathrm{SM})\cdot \tan {\alpha }$ and 95 % CL excluded regions obtained in $H \rightarrow WW^{*} \rightarrow e \nu \mu \nu$ analysis. The expected values are estimated for the signal strength measured in data and assuming best-fit values for all other nuisance parameters. Only data collected at $\sqrt{s}=8$ TeV are used. The symbol “n.a.” denotes the absence of 95 % CL sensitivity

Coupling ratio	Best-fit value	95 % CL exclusion regions	
$H \rightarrow WW^{*} \rightarrow e \nu \mu \nu$	Observed	Expected	Observed
$\tilde{\kappa }_{HVV}/\kappa _\mathrm{SM}$	$-1.3$	$[-1.2, -0.7]$	$(-\infty , -2.2] \bigcup [-1, -0.85] \bigcup [0.4, \infty )$
$(\tilde{\kappa }_{AVV}/\kappa _\mathrm{SM})\cdot \tan {\alpha }$	$-0.2$	n.a.	$(-\infty , -6] \bigcup [5, \infty )$

**Fig. 9:**
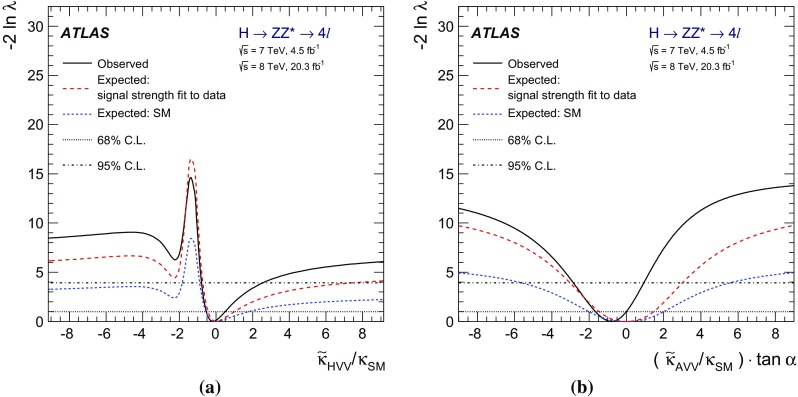
Expected and observed distributions of the test statistic for fits of **a** $\tilde{\kappa }_{HVV}/\kappa _\mathrm{SM}$ and **b** $(\tilde{\kappa }_{AVV}/\kappa _\mathrm{SM})\cdot \tan {\alpha }$ for the $H \rightarrow ZZ^{*} \rightarrow 4 \ell$ analysis. The expected curves are calculated assuming the SM $J^P=0 ^+$ signal and produced with the SM signal strength $\mu =1$ and with the signal strengths fitted to data. The *horizontal dotted black lines* represent the levels of $-2 \ln \lambda$ above which the values of coupling ratios under study are excluded above $68\,\%$ and $95\,\%$ CL, respectively

The contributions of all backgrounds considered in this analysis are also included. By construction the $TO_2$ observables are sensitive to the modulus of the $\tilde{\kappa } _{HVV}/\kappa _\mathrm{SM}$ and $(\tilde{\kappa } _{AVV}/\kappa _\mathrm{SM})\cdot \tan {\alpha }$ ratios: their distributions change with the strength of the respective coupling. These observables are insensitive to the relative sign of $\tilde{\kappa } _{HVV}$ and $\tilde{\kappa } _{AVV}$ with respect to $\kappa _\mathrm{SM}$. The sign sensitivity comes from the $TO_1$ observables, which are based on the interference terms: their distributions feature pronounced sign-dependent asymmetries. It was also found that the observables $TO_1(\tilde{\kappa }_{HVV})$ and $TO_2(\tilde{\kappa }_{HVV})$ are linearly correlated. To maximise the population of analysis histograms with currently available Monte Carlo event samples, it is desirable to reduce this correlation. This is achieved by considering the modified observables $TO_1(\tilde{\kappa }_{HVV}) + TO_2(\tilde{\kappa }_{HVV})$ and $TO_1(\tilde{\kappa }_{HVV}) - TO_2(\tilde{\kappa }_{HVV})$ in the current analysis.

The analysis is performed in several steps. First, multi-dimensional histograms of observables are created in 81 bins of $\tilde{\kappa }_{HVV}/\kappa _\mathrm{SM}$ and $(\tilde{\kappa }_{AVV}/\kappa _\mathrm{SM})\cdot \tan {\alpha }$ for all fits. The predicted shapes of the observables for the signal are produced by reweighting the base Monte Carlo sample described in Sect. 4. The corresponding weights are derived using the analytical calculation of the $H \rightarrow ZZ^{*} \rightarrow 4 \ell$ matrix elements at leading order in perturbative QCD. The weights are calculated and applied at the Monte Carlo generator level. The observables used in the analysis are evaluated after detector simulation, accounting for the detector acceptance, resolution and reconstruction efficiency. The distributions of observables for backgrounds are estimated using Monte Carlo (for the irreducible background) and data-driven techniques (for the reducible backgrounds) described in Sect. 5 and Refs. [12, 18].

The distributions of observables are three-dimensional: $TO_{1}(\tilde{\kappa }_{AVV}$, $\alpha ), TO_{2}(\tilde{\kappa }_{AVV}, \alpha )$, $\mathrm{BDT(}ZZ\mathrm {)}$ and $TO_1(\tilde{\kappa }_{HVV}) + TO_2(\tilde{\kappa }_{HVV})$, $TO_1(\tilde{\kappa }_{HVV}) - TO_2(\tilde{\kappa }_{HVV})$, $\mathrm{BDT(}ZZ\mathrm {)}$ respectively. To obtain a reliable description for bins with an insufficient number of Monte Carlo events, the Kernel Density Estimation [43] smoothing procedure is applied to signal and background multi-dimensional histograms. In the smoothing procedure the smearing is done separately in four bins of $\mathrm{BDT(}ZZ\mathrm {)}$, preserving the original normalisation.

**Table 8:** Expected and observed best-fit values of $\tilde{\kappa }_{HVV}/\kappa _\mathrm{SM}$ and $(\tilde{\kappa }_{AVV}/\kappa _\mathrm{SM})\cdot \tan {\alpha }$ and $95\%$ CL excluded regions obtained in the $H \rightarrow ZZ^{*} \rightarrow 4 \ell$ analysis. The expected values are estimated for the signal strength measured in data and assuming best-fit values for all other nuisance parameters. The data for $\sqrt{s}=7$ TeV and $\sqrt{s}=8$ TeV are combined

Coupling ratio	Best-fit value	95 % CL exclusion regions	
$H \rightarrow ZZ^{*} \rightarrow 4 \ell$	Observed	Expected	Observed
$\tilde{\kappa }_{HVV}/\kappa _\mathrm{SM}$	$-0.2$	$(-\infty , -0.75]\bigcup [6.95, \infty )$	$(-\infty , -0.75] \bigcup [2.45, \infty )$
$(\tilde{\kappa }_{AVV}/\kappa _\mathrm{SM})\cdot \tan {\alpha }$	$-0.8$	$(-\infty , -2.95]\bigcup [2.95, \infty )$	$(-\infty , -2.85] \bigcup [0.95, \infty )$

**Fig. 10:**
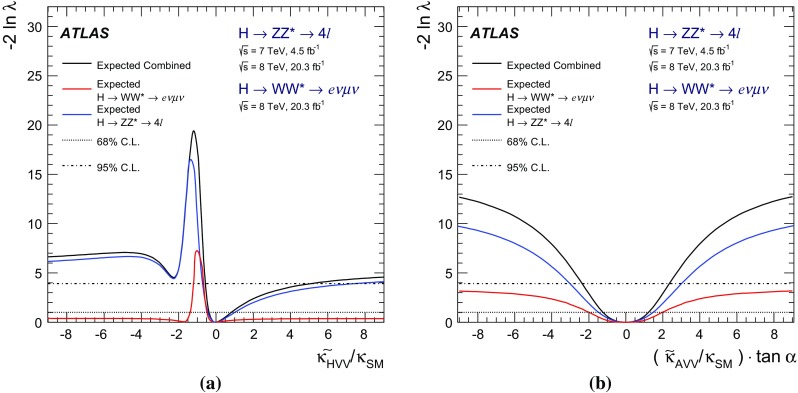
Expected distributions of the test statistic for the combination of $H \rightarrow WW^{*} \rightarrow e \nu \mu \nu$ and $H \rightarrow ZZ^{*} \rightarrow 4 \ell$ analyses as a function of BSM coupling ratios **a** $\tilde{\kappa }_{HVV}/\kappa _\mathrm{SM}$ and **b** $(\tilde{\kappa }_{AVV}/\kappa _\mathrm{SM})\cdot \tan {\alpha }$. The expected values are estimated for the signal strengths measured in data and assuming best-fit values for all other nuisance parameters. The 68 % and 95 % CL exclusion regions are indicated as *lying above the corresponding horizontal lines*. The individual distributions for $H \rightarrow WW^{*} \rightarrow e \nu \mu \nu$ and $H \rightarrow ZZ^{*} \rightarrow 4 \ell$ channels are shown

**Fig. 11:**
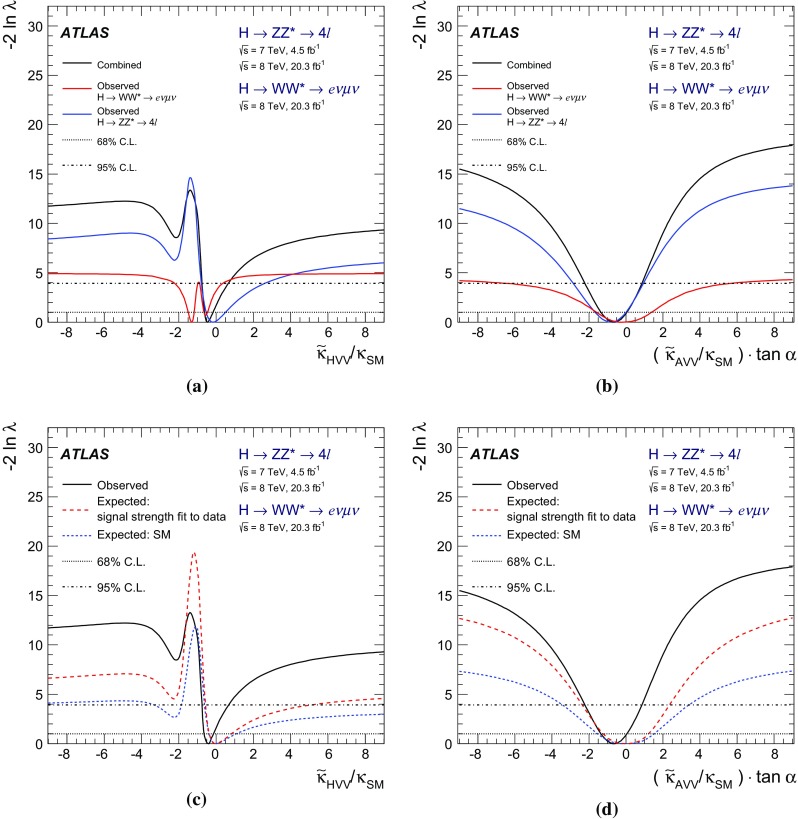
Expected and observed distributions of the test statistic for $H \rightarrow WW^{*} \rightarrow e \nu \mu \nu$ and $H \rightarrow ZZ^{*} \rightarrow 4 \ell$ analyses and their combinations. The distributions are shown as a function of the BSM coupling ratios $\tilde{\kappa }_{HVV}/\kappa _\mathrm{SM}$ and $(\tilde{\kappa }_{AVV}/\kappa _\mathrm{SM})\cdot \tan {\alpha }$. The 68 % and 95 % CL exclusion regions are indicated as *lying above the corresponding horizontal lines*. **a**, **b** Individual $H \rightarrow WW^{*} \rightarrow e \nu \mu \nu$ , $H \rightarrow ZZ^{*} \rightarrow 4 \ell$ and combined observed distributions. **c**, **d** expected and observed combined distributions. The expected distributions are presented for the SM signal strength $\mu =1$ and for the signal strengths obtained from the fit to data

The final pdfs used in the fits are obtained by applying linear histogram interpolation between the multi-dimensional bins of $\tilde{\kappa }_{HVV}/\kappa _\mathrm{SM}$ and $(\tilde{\kappa }_{AVV}/\kappa _\mathrm{SM})\cdot \tan {\alpha }$. The individual likelihood functions per centre-of-mass energy ($\sqrt{s}$) and final state (FS) are:

11 $$\begin{aligned}&\mathcal {L}\left( \bar{\Omega } \Big | \frac{\tilde{\kappa }_{HVV}}{\kappa _\mathrm{SM}}, \frac{\tilde{\kappa }_{AVV}}{\kappa _\mathrm{SM}}\tan {\alpha },\bar{\theta } \right) \nonumber \\&\quad = \prod \limits _{i} P\left[ \bar{ \Omega }_{i} \Big | s_{i} \left( \frac{\tilde{\kappa }_{HVV}}{\kappa _\mathrm{SM}}, \frac{\tilde{\kappa }_{AVV}}{\kappa _\mathrm{SM}}\tan {\alpha } , \bar{\theta } \right) + b_{i}(\bar{\theta }) \right] , \end{aligned}$$

where *P* is the probability density function for the data vector $\bar{\Omega }$, given the signal model *s* and background model *b*. The index *i* runs over all the bins of multi-dimensional histograms of observables and $\bar{\theta }$ represents the vector of nuisance parameters corresponding to systematic uncertainties. Fits to data are performed by minimising the negative log-likelihood function with respect to the ratios of the couplings:

12 $$\begin{aligned}&L\left( \bar{\Omega } \Big | \frac{\tilde{\kappa }_{HVV}}{\kappa _\mathrm{SM}}, \frac{\tilde{\kappa }_{AVV}}{\kappa _\mathrm{SM}}\tan {\alpha }, \bar{\theta } \right) \nonumber \\&\quad = -2 \ln \prod \limits _{\sqrt{s}} \prod \limits _\mathrm{FS} \mathcal {L}\left( \bar{\Omega } \Big | \frac{\tilde{\kappa }_{HVV}}{\kappa _\mathrm{SM}}, \frac{\tilde{\kappa }_{AVV}}{\kappa _\mathrm{SM}}\tan {\alpha }, \bar{\theta } \right) . \end{aligned}$$

The test statistic $q' = -2\ln (\lambda )$ is defined as the profiled value of *L* of Eq. (12). To ensure the correctness of the statistical treatment and the absence of significant biases, a series of tests were performed before applying the fit to the data. Asimov datasets [31, 32] created from independently generated Monte Carlo samples with $\tilde{\kappa }_{HVV} /{\kappa _\mathrm{SM}}$ and $(\tilde{\kappa } _{AVV}/\kappa _\mathrm{SM}) \cdot \tan {\alpha }$ equal to $0, \pm 2, \pm 4, \pm 6, \pm 8$ and $\pm 10$ were injected into the analysis procedure. The tests were repeated for samples corresponding to 1 and 100 times the LHC Run-I integrated luminosity. In all cases the fitted values of coupling constants were found to be in agreement with the injected values within statistical uncertainties.

The results of the matrix-element-observable fit were validated and cross-checked using a nine-dimensional matrix-element method (9D fit). The method implements a multivariate per-event extended likelihood that is sensitive to both the $\tilde{\kappa }_{HVV}/\kappa _\mathrm{SM}$ and $(\tilde{\kappa }_{AVV}/\kappa _\mathrm{SM})\cdot \tan {\alpha }$ mixing parameters and is based on nine experimental observables. The probability model is constructed with separate components for signal, the SM $ZZ^*$ background and the reducible background. The background components are assumed to be independent of the Higgs boson tensor structure, so all of the sensitivity to mixing parameters comes from the signal component. Each component depends on nine experimental observables: $m_{4\ell }$, $p_{\mathrm{T},4\ell }$, $\eta _{4\ell }$, $\cos \theta ^{*}$, $\cos \theta _{1}$, $\cos \theta _{2}$, $\Phi$, $m_{12}$ and $m_{34}$ (described in Sect. 5.4).

**Table 9:** Expected and observed best-fit values of (a) $\tilde{\kappa }_{HVV}/\kappa _\mathrm{SM}$ and (b) $(\tilde{\kappa }_{AVV}/\kappa _\mathrm{SM})\cdot \tan {\alpha }$ and $95\,\%$ CL excluded regions obtained in the combination of $H \rightarrow ZZ^{*} \rightarrow 4 \ell$ and $H \rightarrow WW^{*} \rightarrow e \nu \mu \nu$ analyses. The expected values are estimated for the signal strengths measured in data and assuming best-fit values for all other nuisance parameters. The signal strengths are treated independently per decay channel and per collision energy

Coupling ratio	Best-fit value	95 % CL exclusion regions	
Combined	Observed	Expected	Observed
$\tilde{\kappa }_{HVV}/\kappa _\mathrm{SM}$	$-0.48$	$(-\infty , -0.55]\bigcup [ 4.80, \infty )$	$(-\infty , -0.73]\bigcup [ 0.63, \infty )$
$(\tilde{\kappa }_{AVV}/\kappa _\mathrm{SM})\cdot \tan {\alpha }$	$-0.68$	$(-\infty , -2.33]\bigcup [ 2.30, \infty )$	$(-\infty , -2.18]\bigcup [ 0.83, \infty )$

The main sources of systematic uncertainty for the tensor structure measurements are the same as discussed in Sect. 5 since they are based on the same four-lepton variables. Several additional sources of uncertainty, specific to each of the methods, are also taken into account. For the matrix-element-observable fit, the uncertainty related to the Kernel Density Estimation smoothing procedure applied to signal and background multi-dimensional histograms is considered. To estimate the influence of this uncertainty on the final result, a procedure similar to the one described in Sect. 5 is employed. The impact of the different sources of systematic uncertainty on the final results is evaluated by comparing the BSM exclusion limits obtained with a specific systematic uncertainty included or excluded in the fit, while excluding all other systematic uncertainties. A similar conclusion holds in the fixed hypothesis test: the systematic uncertainties have a very limited impact on the final result. The most important uncertainties are related to the estimates of the reducible backgrounds. The relative impact of these uncertainties on the final 95 % CL exclusion limit on BSM couplings was found to be around $\pm 1\,\%$. The second most important group of sources of systematic uncertainty is related to the theoretical uncertainties on the production cross section of the $ZZ^{*}$ background process. Their relative impact on the final result is found to be less than $\pm 1\,\%$. The precision of the tensor structure analysis is thus dominated by the statistical errors.

In this paper, only results based on the matrix-element-observable approach are reported. The 9D approach was used as a cross-check and produced results compatible with the matrix-element approach.

### Individual and combined results

The results of the tensor structure analyses performed in the $H \rightarrow WW^{*} \rightarrow e \nu \mu \nu$ channel are reported in Ref. [8] and, for completeness, they are also summarised in Table 7.

The distributions of the test statistic for fits of $\tilde{\kappa }_{HVV}/\kappa _\mathrm{SM}$ and $(\tilde{\kappa }_{AVV}/\kappa _\mathrm{SM})\cdot \tan {\alpha }$ measured in the $H \rightarrow ZZ^{*} \rightarrow 4 \ell$ analysis are shown in Fig. 9.

The expected curves are calculated assuming the SM $J^P=0 ^+$ signal, both with the SM signal strength, $\mu =1$, and with the signal strength fitted to data, $\hat{\mu }$. The fitted values of $\tilde{\kappa }_{HVV}/\kappa _\mathrm{SM}$ and $(\tilde{\kappa }_{AVV}/\kappa _\mathrm{SM})\cdot \tan {\alpha }$, together with the intervals where these couplings are excluded at above the $95\%$ CL, are reported in Table 8. The fitted values agree with the SM predictions within uncertainties.

The measurements from the $H \rightarrow WW^{*} \rightarrow e \nu \mu \nu$ and $H \rightarrow ZZ^{*} \rightarrow 4 \ell$ channels are combined under the assumption that the BSM ratios of couplings $\tilde{\kappa }_{HVV}/\kappa _\mathrm{SM}$ and $(\tilde{\kappa }_{AVV}/\kappa _\mathrm{SM})\cdot \tan {\alpha }$ are the same for the *W* and *Z* vector bosons. A common test statistic is obtained by combining the profiled likelihoods of the individual channels. The expected distributions of the likelihoods, for the signal strength values obtained from the fits to the data ($\mu = \hat{\mu }$), are presented in Fig. 10.

The observed distributions of profiled likelihoods for the combination of $H \rightarrow WW^{*} \rightarrow e \nu \mu \nu$ and $H \rightarrow ZZ^{*} \rightarrow 4 \ell$ measurements are presented in Fig. 11. The asymmetric shape of the expected and observed limits in the $\tilde{\kappa }_{HVV}/\kappa _\mathrm{SM}$ results is mainly due to the interference between the BSM and the SM contributions that gives maximum deviation from the SM predictions for negative relative values of the BSM couplings.

Here the signal normalisations are treated as independent nuisance parameters of the different decay channels and the different centre-of-mass energies. The other nuisance parameters related to the experimental and theoretical uncertainties are treated as correlated when appropriate. The resulting 95 % CL exclusion regions for the combinations of $H \rightarrow WW^{*} \rightarrow e \nu \mu \nu$ and $H \rightarrow ZZ^{*} \rightarrow 4 \ell$ channels are listed in Table 9.

## Conclusion

Studies of the spin and parity of the observed Higgs boson in the $H \rightarrow ZZ^{*} \rightarrow 4 \ell$, $H \rightarrow WW^{*} \rightarrow e \nu \mu \nu$ and $H \rightarrow \gamma \gamma$ decay processes are presented. The investigations are based on 4.5 and $20.3\;\mathrm{fb}^{-1}$ of *pp* collision data collected by the ATLAS experiment at the LHC at $\sqrt{s}=7$ TeV and $\sqrt{s}=8$ TeV, respectively. The SM Higgs boson hypothesis, corresponding to the quantum numbers $J^{P}=0^{+}$, is tested against several alternative spin and parity models. The models considered include non-SM spin-0 and spin-2 models with universal and non-universal couplings to quarks and gluons. The combination of the three decay processes allows the exclusion of all considered non-SM spin hypotheses at a more than 99.9 % CL in favour of the SM spin-0 hypothesis.

The tensor structure of the *HVV* interaction in the spin-0 hypothesis is also investigated using the $H \rightarrow ZZ^{*} \rightarrow 4 \ell$ and $H \rightarrow WW^{*} \rightarrow e \nu \mu \nu$ decays. Only one BSM tensor coupling is investigated at a time, while the other one is set to zero. The observed distributions of the variables sensitive the ratios of the BSM to SM tensor couplings, $\tilde{\kappa }_{HVV}/\kappa _\mathrm{SM}$ and $(\tilde{\kappa }_{AVV}/\kappa _\mathrm{SM})\cdot \tan {\alpha }$, are compatible with the SM predictions.

Values of the BSM tensor couplings outside of the intervals $-0.75< \tilde{\kappa }_{HVV}/\kappa _\mathrm{SM}< 2.45$ and $-2.85< (\tilde{\kappa }_{AVV}/\kappa _\mathrm{SM})\cdot \tan {\alpha }< 0.95$ are excluded at the 95 % CL for the $H \rightarrow ZZ^{*} \rightarrow 4 \ell$ process. For the $H \rightarrow WW^{*} \rightarrow e \nu \mu \nu$ process the ranges $-2.2< \tilde{\kappa }_{HVV}/\kappa _\mathrm{SM}< -1.0$ and $-0.85< \tilde{\kappa }_{HVV}/\kappa _\mathrm{SM}< 0.4$ and $-6.0< (\tilde{\kappa }_{AVV}/\kappa _\mathrm{SM})\cdot \tan {\alpha }< 5.0$ are excluded at the 95 % CL.

The results from the $H \rightarrow WW^{*} \rightarrow e \nu \mu \nu$ and $H \rightarrow ZZ^{*} \rightarrow 4 \ell$ decay channels are combined under the assumption that the $\tilde{\kappa }_{HVV}/\kappa _\mathrm{SM}$ and $(\tilde{\kappa }_{AVV}/\kappa _\mathrm{SM})\cdot \tan {\alpha }$ couplings have the same values for the *HWW* and *HZZ* processes. As a result of this combination, the regions outside of $-0.73< \tilde{\kappa }_{HVV}/\kappa _\mathrm{SM}< 0.63$ and $-2.18< (\tilde{\kappa }_{AVV}/\kappa _\mathrm{SM})\cdot \tan {\alpha }< 0.83$ intervals are excluded at the 95 % CL. The corresponding expected not-excluded intervals at the 95 % CL, assuming the SM Higgs boson hypothesis and the signal strength values measured in data, are $-0.55< \tilde{\kappa }_{HVV}/\kappa _\mathrm{SM}< 4.80$ and $-2.33< (\tilde{\kappa }_{AVV}/\kappa _\mathrm{SM})\cdot \tan {\alpha }< 2.30$.

## Appendix A

To compare the exclusion limits obtained in this analysis to other existing studies, the final results of this analysis are also expressed in terms of effective cross-section fractions $(f_{g2},\phi _{g2})$ and $(f_{g4},\phi _{g4})$. The definitions proposed in Section 11.4.2 of Ref. [3] and Section II of Ref. [44] are used:

13 $$\begin{aligned} f_{gi}=\frac{|g_i|^2 \sigma _i}{|g_1|^2 \sigma _1 + |g_2|^2 \sigma _2 + |g_4|^2 \sigma _4},\quad \phi _i =\arg {\left( \frac{g_i}{g_1} \right) }.\nonumber \\ \end{aligned}$$

Here the symbols $g_1$, $g_2$ and $g_4$ denote the SM, BSM CP-even and BSM CP-odd tensor couplings of the *HVV* scattering amplitude, respectively. The numeric coefficients $\sigma _1$, $\sigma _2$ and $\sigma _4$ are effective cross sections of the *HVV* interactions calculated when only the $g_1$-, $g_2$- or $g_4$-related terms are present in the amplitude, respectively, such that $g_i=1,g_{i \ne j} =0$.

**Table 10:** Expected limits on $(f_{g2},\phi _{g2})$ and $(f_{g4}, \phi _{g4})$ parameters defined in Ref. [3] obtained in the analyses of the $H \rightarrow WW^{*} \rightarrow e \nu \mu \nu$ and $H \rightarrow ZZ^{*} \rightarrow 4 \ell$ channels and for their combination. The symbol “n.a.” denotes the absence of 95 % CL sensitivity

Expected $95\%$ CL limits	
$$H \rightarrow WW^{*} \rightarrow e \nu \mu \nu $$	
n.a. for $\phi _{g2} = 0$ and $f_{g2}<0.15; f_{g2}>0.33$ for $\phi _{g2} = \pi$	
n.a. for $\phi _{g4} = 0$ and n.a. for $\phi _{g4} = \pi$	
$$H \rightarrow ZZ^{*} \rightarrow 4 \ell $$	
$f_{g2}<0.94$ for $\phi _{g2} = 0$ and $f_{g2}<0.16$ for $\phi _{g2} = \pi$	
$f_{g4}<0.56$ for $\phi _{g4} = 0$ and $f_{g4}<0.56$ for $\phi _{g4} = \pi$	
Combination of $H \rightarrow ZZ^{*} \rightarrow 4 \ell$ and $H \rightarrow WW^{*} \rightarrow e \nu \mu \nu$	
$f_{g2}<0.89$ for $\phi _{g2} = 0$ and $f_{g2}<0.096$ for $\phi _{g2} = \pi$	
$f_{g4}<0.43$ for $\phi _{g4} = 0$ and $f_{g4}<0.44$ for $\phi _{g4} = \pi$

**Table 11:** Observed imits on $(f_{g2},\phi _{g2})$ and $(f_{g4}, \phi _{g4})$ parameters defined in Ref. [3] obtained in the analyses of the $H \rightarrow WW^{*} \rightarrow e \nu \mu \nu$ and $H \rightarrow ZZ^{*} \rightarrow 4 \ell$ channels and for their combination

Observed 95 % CL limits	
$$H \rightarrow WW^{*} \rightarrow e \nu \mu \nu $$	
$f_{g2}<0.053$ for $\phi _{g2} = 0$ and $f_{g2}<0.20;\; 0.26<f_{g2}<0.63$ for $\phi _{g2} = \pi$	
$f_{g4}<0.78$ for $\phi _{g4} = 0$ and $f_{g4}<0.84$ for $\phi _{g4} = \pi$	
$$H \rightarrow ZZ^{*} \rightarrow 4 \ell $$	
$f_{g2}<0.68$ for $\phi _{g2} = 0$ and $f_{g2}<0.16$ for $\phi _{g2} = \pi$	
$f_{g4}<0.11$ for $\phi _{g4} = 0$ and $f_{g4}<0.54$ for $\phi _{g4} = \pi$	
Combination of $H \rightarrow ZZ^{*} \rightarrow 4 \ell$ and $H \rightarrow WW^{*} \rightarrow e \nu \mu \nu$	
$f_{g2}<0.12$ for $\phi _{g2} = 0$ and $f_{g2}<0.16$ for $\phi _{g2} = \pi$	
$f_{g4}<0.090$ for $\phi _{g4} = 0$ and $f_{g4}<0.41$ for $\phi _{g4} = \pi$	

When, in addition to the SM term, only one CP-even or CP-odd BSM contribution is present, the conversion between the parameterisation used in this analysis and the $(f_{gi},\phi _{gi})$ parameterisation is given by Eq. (13) rewritten in the following way:

14 $$\begin{aligned} f_{g_i}=\frac{r_{i1}^2}{1+r_{i1}^2}; \quad (i=2,4), \end{aligned}$$

where $r_{41}$ and $r_{21}$ are chosen such that:

15 $$\begin{aligned} r_{21}^2= & {} \frac{\sigma _{HVV}}{\sigma _\mathrm{SM}} \left( \frac{ \tilde{k}_{HVV}}{k_\mathrm{SM}} \right) ^2, \quad \mathrm{and} \nonumber \\ r_{41}^2= & {} \frac{\sigma _{AVV}}{\sigma _\mathrm{SM}} \left( \frac{ \tilde{k}_{AVV}}{k_\mathrm{SM}} \right) ^2 \tan ^2{\alpha }. \end{aligned}$$

The numeric coefficients $\sigma _\mathrm{SM}$, $\sigma _{HVV}$ and $\sigma _{AVV}$ are effective cross sections of the *HVV* interaction calculated when only each of the $\kappa _\mathrm{SM}$-, $\kappa _{HVV}$- and $\kappa _{AVV}$-related terms is present in the Lagrangian.

For consistency with previous measurements reported in Ref. [5], the expected and observed results of the current analysis of the $H \rightarrow WW^{*} \rightarrow e \nu \mu \nu$ and $H \rightarrow ZZ^{*} \rightarrow 4 \ell$ channels and for their combination are expressed in terms of $f_{gi}$ and $\phi _{gi}$ parameters for the $H \rightarrow ZZ^{*} \rightarrow 4 \ell$ decay, $(f_{g2}^{ZZ} ,\phi _{g2} ^{ZZ})$ and $(f_{g4}^{ZZ},\phi _{g4}^{ZZ})$. These parameters are denoted hereafter by $(f_{g2},\phi _{g2})$ and $(f_{g4}, \phi _{g4})$. The corresponding results are presented in Tables 10 and 11.

To obtain these results, the effective cross sections $\sigma _\mathrm{SM}$, $\sigma _{HVV}$ and $\sigma _{AVV}$ of the *HZZ* interaction are calculated using the MadGraph5_aMC@NLO Monte Carlo generator [16] at leading order. The ratios of cross sections used in the calculation are: $\sigma _{HVV}/\sigma _\mathrm{SM} = 0.349$ and $\sigma _{AVV}/\sigma _\mathrm{SM} = 0.143$, respectively.

## Footnotes

1 In the following, for brevity, only the $J^P$ label is used to indicate the spin and CP quantum numbers.

2 In this paper the symbol *V* is used to describe a massive SM vector boson, namely either a *W* or a *Z* boson.

3 Here channel can be used to indicate different categories in the same final state when producing results for individual decay channels, or different final states when combining them.

4 Throughout this section, the following variables are used: $p_{\text {T}} ^{\ell \ell }$ and $m_{\ell \ell }$ are the transverse momentum and the invariant mass of the two-lepton system, respectively, $\Delta \phi _{\ell \ell }$ is the azimuthal angular difference between the two leptons, $m_\mathrm{T}$ is the transverse mass of the reconstructed Higgs boson decay system, $\Delta p_\mathrm{T}$ is the absolute value of the difference between the momenta of the two leptons and $E_{\ell \ell \nu \nu } =p_{\text{ T }}^{{\ell }_1} -0.5p_{\text{ T }}^{{\ell }_2}+0.5 p_{\text{ T }}^{miss}$, where $p_{\text{ T }}^{miss}$ is the missing transverse momentum.
